# Recommendation admissibility for finite computational benchmarks under empirical-resampling variability and aggregation-design uncertainty

**DOI:** 10.1016/j.mex.2026.104238

**Published:** 2026-11-02

**Authors:** Krisztián Attila Bakon

**Affiliations:** University of Pannonia, Nagykanizsa – University Center for Circular Economy, Zrínyi Miklós u. 18, Nagykanizsa, 8800, Hungary

**Keywords:** Computational benchmarking, Recommendation admissibility, Empirical resampling, Aggregation-design uncertainty, Evidence governance, Flexible job shop scheduling

## Abstract

Computational benchmarks may identify a point winner even when the represented evidence does not justify releasing a stronger recommendation. C7 is an executable protocol for deciding whether a bounded algorithm recommendation is admissible on a declared finite computational-benchmark evidence surface. It evaluates two distinct components separately: empirical-resampling variability under a frozen perturbation contract and aggregation-design uncertainty over a prespecified admissible design set. Complete-portfolio support and aggregation-design behavior are then combined through the A1–A5 release grammar, with mandatory evidence or authority failures handled by fail-closed gates. In the FJSP demonstration, V1 retains MWKR as the point recommendation but no candidate achieves complete support, yielding A3 — Sampling-inconclusive. In V2, ECT is point-recommended and supported at both required endpoints; under the additive/common-calibration geometry, that support extends across the declared segment, yielding A1 — Supported and aggregation-design-stable. These conclusions remain limited to the declared finite surfaces, matched portfolios, represented conditions, replication structures, and admissible aggregation-design domains; they do not establish universal solver superiority, future-instance inference, deployment validity, or certification.

## Graphical abstract

**Figure:**
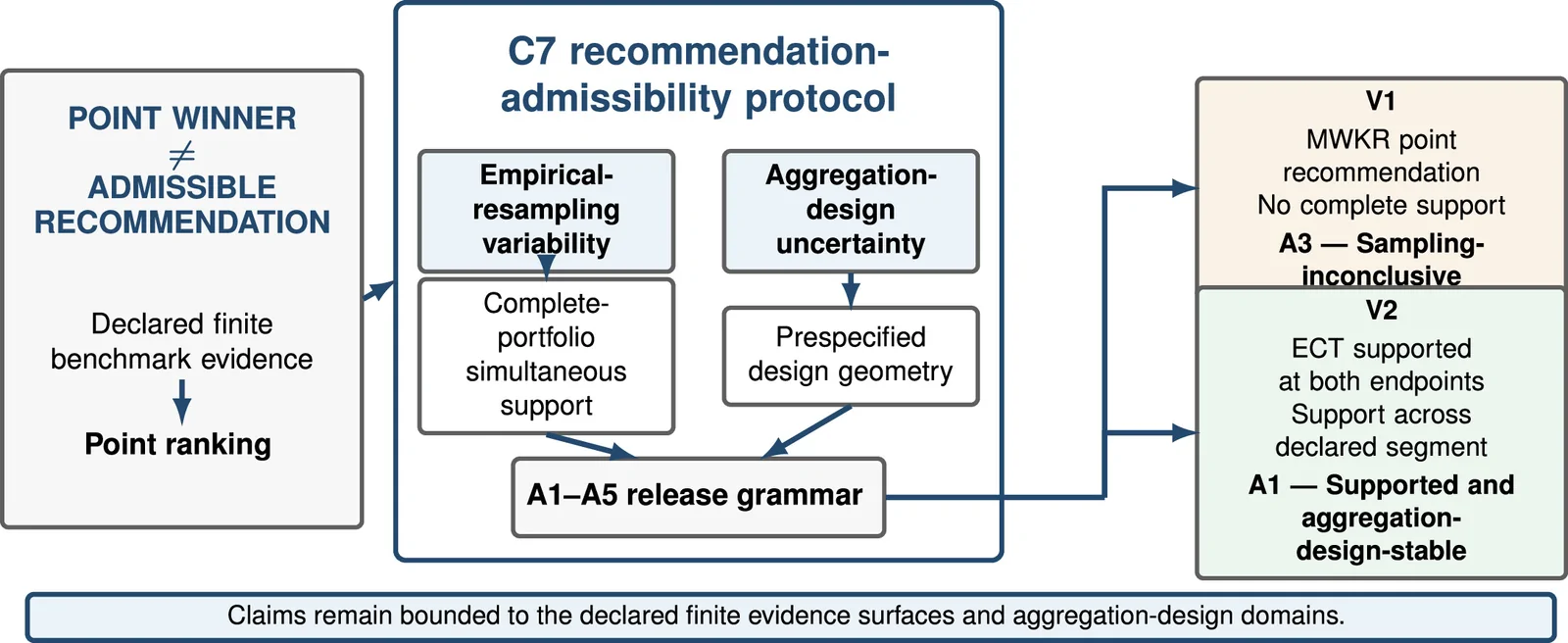

• C7 governs release of bounded recommendations rather than point rankings alone.
• It evaluates empirical-resampling variability and aggregation-design uncertainty separately.
• Complete-portfolio support, design geometry, and fail-closed gates determine release states.

**Table:** 

**Specifications table: C7 recommendation-admissibility protocol**	
Field	Specification
Subject area	Computer Science
More specific subject area	Computational benchmarking and algorithm evaluation
Name of method	C7 recommendation-admissibility protocol
Name/reference of original method	N/A. C7 integrates established comparison, empirical-resampling, aggregation-design,
	and provenance components into a bounded recommendation-release rule; it does not
	claim a single parent original method.
Resource availability	Supporting evidence, code, schemas, contracts, derived artifacts, provenance, and
	integrity material: public Figshare evidence-support package,
	https://doi.org/10.6084/m9.figshare.33287469.
	Raw third-party benchmark source files are not redistributed.

## Background

Computational benchmarks can identify a point winner without establishing that finite represented evidence warrants a stronger recommendation. A point ranking records the best score under a specified aggregation; recommendation admissibility is stronger because the evidential and design conditions for releasing that claim must also be satisfied. The target is a declared finite evidence surface, not a population draw or a solver leaderboard.

Two distinct axes govern that decision. Empirical-resampling variability concerns represented finite evidence under an authorized perturbation mechanism, with the benchmark surface, matched portfolio, and target weights fixed. Aggregation-design uncertainty concerns recommendation behavior across a prespecified set of scientifically interpretable target-weight conventions. Resampling varies represented evidence at fixed design, whereas aggregation design varies target weights over the declared design set; neither axis substitutes for the other.

C7 links established comparison, resampling, aggregation, sensitivity, and provenance components to one executable, claim-bounded release decision. It forms matched portfolio-relative scores, applies complete-portfolio empirical-resampling support at prespecified designs that enter the A1–A5 classification, and evaluates aggregation-design behavior under the A1–A5 precedence and mandatory evidence gates. The contribution is this integration, not new component mathematics. The research question is: On a declared finite benchmark surface with a prespecified admissible aggregation-design set, when may a bounded algorithm recommendation be released after empirical-resampling variability and aggregation-design uncertainty have been evaluated separately under the same claim rule?

Flexible job shop scheduling (FJSP) is the demonstration domain, not the contribution class. V1 and V2 are separately scoped finite FJSP surfaces, not pooled samples or a validation hierarchy. Claims remain indexed to their declared surfaces, portfolios, conditions, replication structures, aggregation-design domains, and release grammar; they do not establish general solver superiority, superpopulation or future-instance inference, deployment validity, or certification.

## Method details

### Declared finite evidence surface and matched portfolio

Throughout C7, “claim-bearing” refers to evidence or requirements that authorize or support a released claim, whereas “class-bearing” refers to prespecified designs or results that enter the A1–A5 classification.

C7 begins from the scientific target rather than from a resampling distribution. Let D denote a declared, authorized finite computational benchmark evidence surface. Its claim-bearing content comprises the represented benchmark cases and conditions, their fixed structural and group definitions, the authorized repeated-evidence identities, a matched algorithm portfolio A, and the admissible aggregation-design conventions under which the represented evidence may be summarized. A claim-bearing matched cell *c* contains the authorized outcome for every a∈A under the same case, condition, and repeated-evidence identity. The target is exactly this represented finite surface under its declared contracts. It is not treated as a probability sample from all possible problem instances, manufacturing systems, deployment environments, or another unobserved superpopulation. Resampling may perturb only what its frozen contract authorizes; it does not enlarge that target.

Let *y*_*c,a*_ be the lower-is-better performance value for algorithm *a* in matched cell *c*. The frozen cell-level comparison is the portfolio-relative normalized regret

$$r_{c,a}=\frac{y_{c,a}-\min_{b\in A}y_{c,b}}{\min_{b\in A}y_{c,b}}.$$

If the within-cell best objective $\min_{b\in A}y_{c,b}$ is nonpositive, the entire matched cell is excluded for all algorithms under the frozen contract, and that exclusion must be recorded. An unrecorded or unauthorized exclusion is a mandatory evidence failure. No algorithm-specific denominator or silent renormalization is permitted. The denominator is otherwise the best observed member of the declared portfolio in the same authorized cell, so every member attaining the within-cell minimum has zero regret. The comparison is therefore matched within cells and relative to A. It is not an exact-optimum, oracle, or certified-optimality gap, and changing the declared portfolio changes the estimand.

Every surface contract explicitly fixes its within-group operator before outcome interpretation; it cannot remain unspecified or be selected after results are observed. In both demonstrations, *R*_*g,a*_ is the arithmetic mean of all authorized matched cells in group *g* after the whole-cell exclusion rule, using the same authorized cell basis for every algorithm. No whole-cell exclusion was exercised on either claim-bearing surface because every authorized within-cell minimum was positive. For V1, with group g=(s,p), represented-instance set *I_s_*, $n_{s}=\left|I_{s}\right|$, preset *p*, and replication ID *h*,

$$R_{(s,p),a}=\frac{1}{10n_{s}}\sum_{i\in I_{s}}\sum_{h=0}^{9}r_{(i,p,h),a}.$$

For V2, whose structural stratum contains five represented instances,

$${\bar{R}}_{i,p,a}=\frac{1}{10}\sum_{h=0}^{9}r_{(i,p,h),a},\;R_{(s,p),a}=\frac{1}{5}\sum_{i\in I_{s}}{\bar{R}}_{i,p,a}.$$

Thus the V2 group operator is also the arithmetic mean over its 5×10=50 authorized matched cells. Completeness of the matched portfolio and validity of required denominator/evidence records are claim-bearing requirements rather than opportunities for algorithm-specific imputation or silent renormalization.

### Portfolio-relative outcome and point recommendation

An aggregation design is a nonnegative group-weight vector $w=(w_{g}:g\in G)$ with $\sum_{g}w_{g}=1$, interpreted according to a declared benchmark target convention. The design score is

$$\theta_{a}\left(w\right)=\sum_{g\in G}w_{g}R_{g,a},$$

and the point recommendation is the set

$$D\left(w\right)=\arg\min_{a\in A}\theta_{a}\left(w\right).$$

The recommendation is deliberately set-valued: a tie remains a tie for scientific purposes, and no lexical, file-order, or implementation tie-break may create a unique claim-bearing recommendation. The fixed authorized evidence surface is denoted D, while *D*(*w*) is reserved for this set-valued point-recommendation map.

For any ordered pair a,b∈A, define the directional margin

$$M_{a,b}\left(w\right)=\theta_{b}\left(w\right)-\theta_{a}\left(w\right)=\sum_{g\in G}w_{g}\left(R_{g,b}-R_{g,a}\right).$$

Positive *M*_*a,b*_(*w*) favors candidate *a*, zero is the point-recommendation tie boundary between *a* and *b*, and a negative value favors *b*. A reference design *w*_0_ is a declared target convention used to anchor the reference recommendation; it is not an objectively privileged weighting. It belongs to $B_{\text{adm}}$ and is a prespecified directly calibrated class-bearing design included in the simultaneous coordinate family.

### Two distinct decision axes

C7 separates two decision axes that act on different objects. Empirical-resampling variability is evaluated through the frozen conditional two-component empirical-resampling calibration: authorized empirical multiplicities are perturbed on the already represented fixed surface while an aggregation design is held fixed. It concerns how the class-bearing margins behave under that declared perturbation mechanism. Aggregation-design uncertainty concerns how the point and supported recommendation behave when the target aggregation convention moves within a prespecified scientifically admissible design domain. The first axis perturbs empirical representation under a frozen resampling contract; the second varies declared target weights. They are not pooled into one uncertainty measure, and aggregation designs are not bootstrap draws or random preferences.

This separation also fixes the role of descriptive outputs. Conditional empirical-resampling recommendation frequencies may summarize how often a point recommendation appears under the resampling procedure, but they are not support probabilities or class thresholds. Likewise, aggregation sensitivity cannot establish evidential support by itself. C7 combines the two axes only through the release grammar after each has been evaluated according to its own object. The protocol therefore does not use one axis as a proxy for the other.

### Conditional empirical-resampling calibration and simultaneous support

Conditional on the fixed authorized surface D, the frozen two-component empirical-resampling contract induces

$$Q^{*}\left(\cdot |D\right),$$

the conditional empirical-resampling reference distribution. The benchmark corpus, structural and group definitions, algorithm identities and pairings, and target aggregation weights remain fixed. Instance resampling changes empirical multiplicities and hence the composition of already represented cases within the fixed strata. Replication-ID resampling changes the empirical multiplicities of authorized repeated-evidence identities under the frozen coupling rules. The target aggregation weights remain fixed throughout: bootstrap multiplicities never redefine benchmark target mass or the admissible aggregation design. No iid all-problems or manufacturing superpopulation is introduced, and the construction is not design-based finite-population inference. Its probability semantics are conditional on the represented evidence and the frozen perturbation contract.

For V1 resample *r*, if $I_{r,s,1},\ldots ,I_{r,s,n_{s}}$ are the resampled instance identities and $H_{r,s,1},\ldots ,H_{r,s,10}$ the resampled replication identities in stratum *s*, the implemented group score is

$$R_{r,(s,p),a}^{*}=\frac{1}{10n_{s}}\sum_{j=1}^{n_{s}}\sum_{\ell =1}^{10}r_{(I_{r,s,j},p,H_{r,s,\ell }),a}.$$

For V2 the corresponding implemented score is

$$R_{r,(s,p),a}^{*}=\frac{1}{50}\sum_{j=1}^{5}\sum_{\ell =1}^{10}r_{(I_{r,s,j},p,H_{r,s,\ell }),a}.$$

Duplicate instance identities and duplicate replication IDs contribute according to their sampled multiplicities. Within each frozen stratum, the same draw vectors are applied jointly across represented presets and all five algorithms, preserving matched coupling. These multiplicities do not alter the aggregation target weights.

Let *k* index the complete prespecified simultaneous family of ordered pairwise margin coordinates at the class-bearing fixed designs or vertices required by a surface contract. Both ordered directions are retained at every required design. Write *M_k_* for the point margin and $M_{r,k}^{*}$ for the corresponding margin in empirical resample *r*. The frozen ordered-directional max-deviation implementation is

$$\delta_{r,k}=M_{k}-M_{r,k}^{*},\;z_{r}=\max_{k}\delta_{r,k},$$

followed by

$$c_{0.95}=q_{0.95}^{\text{linear}}\left\{z_{1},\ldots ,z_{R}\right\},\;R=20,000,$$

For sorted values *z*_(1)_ ≤ ⋅⋅⋅ ≤ *z*_(*R*)_, the frozen NumPy quantile(..., method=“linear”) convention uses h=(R−1)p, j=⌊h⌋, and γ=h−j, where *j* is zero-based:

$$q_{p}^{\text{linear}}=\left(1-\gamma \right)z_{\left(j+1\right)}+\gamma z_{\left(j+2\right)}.$$

At R=20,000 and p=.95, h=18,999.05, so

$$c_{0.95}=z_{\left(19000\right)}+0.05\left[z_{\left(19001\right)}-z_{\left(19000\right)}\right].$$

and

$$B_{k}=M_{k}-c_{0.95}.$$

For each ordered pair, $M_{b,a}\left(w\right)=-M_{a,b}\left(w\right)$, and the corresponding empirical-resampling deviations therefore have opposite signs. Because both directions are retained, the maximum over the complete ordered family is algebraically equal to the maximum absolute deviation over one representative of each unordered pair/design. The implementation nevertheless uses the prespecified ordered family, $z_{r}=\max_{k}\delta_{r,k}$, without removing coordinates after observing the results. Consequently,

$$z_{r}\leq c_{0.95}\;⟺\;M_{r,k}^{*}\geq M_{k}-c_{0.95}=B_{k}$$

for every frozen coordinate *k*.

The critical value *c*_0.95_ is the linear empirical 0.95 quantile of the conditional empirical-resampling *z_r_* values. The value 0.95 is the prespecified nominal empirical-resampling decision-calibration level of the frozen reference procedure. It is not frequentist confidence coverage, design-based finite-population coverage, the probability that a candidate is truly best, or a future-instance success probability. The quantity *B_k_* is the simultaneous resampling-calibrated lower bound for coordinate *k*. One common max-deviation subtraction is formed over the complete declared coordinate family; the procedure is not narrowed after a preferred candidate or challenger is observed.

At each prespecified class-bearing fixed design *w* included in the frozen simultaneous coordinate family, candidate *a* is resampling-supported only if every required simultaneous resampling-calibrated lower margin against the complete declared portfolio is strictly positive:

$$\mathrm{Supp}\left(a,w\right)=1\;⟺\;B_{k(a,b,w)}>0\;\text{for}\;\text{every}\;b\in A\setminus \left\{a\right\}.$$

This directly calibrated support predicate is evaluated only at those prespecified class-bearing designs. For an additive convex domain, interior support may follow from the frozen linear-margin and common-calibration geometry when the required vertex conditions imply that all relevant lower margins remain strictly positive; no new bootstrap coordinate or calibration is formed for an arbitrary interior point. The complete family is essential because the released statement concerns candidate *a* relative to the declared portfolio, not relative to a selected challenger. Family membership remains frozen before result interpretation, including authorized reverse-pair and nonreference coordinates. A smallest point score, uncoordinated pairwise summaries, or a high recommendation frequency cannot replace the strict complete-portfolio predicate. Each resample therefore contributes one maximum deviation over the full authorized family and the common subtraction applies to every coordinate. C7 makes no additional coverage theorem or multiple-testing claim beyond that prespecified calibration.

### Admissible aggregation-design set and recommendation-changing boundary

Aggregation-design uncertainty is evaluated only over the prespecified admissible domain. For an additive design this may be written

$$B_{\text{adm}}=\mathrm{conv}\left\{w^{\left(1\right)},\ldots ,w^{\left(K\right)}\right\},$$

where each vertex is a complete, scientifically interpretable benchmark target convention. The set is not a probability distribution over analyst preferences, and it is not enlarged to an unrestricted simplex merely to find a reversal. Its vertices, constraints, and scientific meanings are part of the declared evidence target and remain fixed during empirical resampling.

For a unique reference candidate $D\left(w_{0}\right)=\left\{a_{0}\right\}$, the nearest admissible recommendation-changing boundary is defined, when such a tie exists, by

$$\rho =\min_{b\neq a_{0}}\;\min_{\begin{array}{c} w\in B_{\text{adm}}:\;M_{a_{0},b}\left(w\right)=0 \end{array}}d\left(w,w_{0}\right),$$

with *d* the frozen design-space distance. The search is over every declared challenger and only over $B_{\text{adm}}$. If no admissible challenger tie exists, the protocol reports BOUNDARY_OUTSIDE_B_ADM; it does not fabricate a distance in an unrestricted simplex or another design domain. If decision-critical geometry cannot be resolved under the authorized contract, the boundary is unresolved. Solver tolerances, nonunique optimal-face handling, and implementation mechanics are supplementary details. Boundary stability is a property of the declared aggregation geometry and is distinct from empirical-resampling support at fixed designs.

### A1–A5 recommendation-admissibility grammar

C7 returns exactly one primary recommendation-admissibility class. The frozen precedence is

$$\begin{array}{c} A5\;\to \;A3\;\to \;A1\;\to \;A2\;\to \;A4 \end{array}.$$

The first triggered rule determines the released class. A5 is fail-closed: a missing, unauthorized, inconsistent, or otherwise invalid mandatory claim-bearing evidence or authority requirement prevents stronger release. After those mandatory gates pass, A3 — Sampling-inconclusive applies when the reference point recommendation is tied, no candidate clears the complete-portfolio support predicate at the prespecified directly calibrated class-bearing *w*_0_, or a decision-critical reference margin remains unresolved. Here, ’Sampling-inconclusive’ is the fixed class label; it denotes inconclusiveness under the frozen empirical-resampling support calibration, not probability-sample inference.

If A5 and A3 do not trigger and a unique reference candidate is resampling-supported, A1, A2, and A4 distinguish the aggregation-design state. A1 — Supported and aggregation-design-stable requires the same candidate to satisfy the support rule at every distinct required vertex of the declared additive $B_{\text{adm}}$. Under the frozen additive geometry, the linear margins and common calibration carry strict vertex support through that convex domain; the resulting stability statement is only over $B_{\text{adm}}$. A2 — Supported but aggregation-design-conditional applies when A1 is false, the reference candidate is supported at *w*_0_, at least one different candidate is resampling-supported at least one distinct required admissible vertex, and a resolved recommendation-changing boundary exists inside $B_{\text{adm}}$. Arbitrary interior support is not a separate A2 trigger. A4 — Aggregation-design-inconclusive applies when the reference candidate is supported but neither A1 nor A2 is established after complete frozen design and boundary evaluation, leaving the status across $B_{\text{adm}}$ unresolved.

Table 1 states this grammar compactly. The classes are release states rather than performance tiers, and no class is assigned from a point ranking alone or from absence of an observed reversal.

**Table 1:** A1–A5 recommendation-admissibility grammar.

Class	Precedence trigger	Support/design state	Permitted release	Key boundary / interpretive restriction
**A5**	Evaluated first: a mandatory claim-bearing evidence or authority requirement is missing, unauthorized, inconsistent, or invalid.	Evidence is not authorized for class release.	Name the failed requirement; release no recommendation-admissibility claim.	Fail closed before stronger release. The failure is not empirical-resampling variability and cannot be repaired by outcome-dependent contract changes.

**A3 — Sampling-inconclusive**	A5 did not trigger and the reference recommendation is tied, no candidate clears complete resampling support at *w*_0_, or a decision-critical reference margin remains unresolved.	Reference-design support is absent or unresolved under the complete calibration.	Report any resolved point recommendation descriptively; do not release a supported recommendation at the reference design.	Sampling is the frozen label: inconclusive under the frozen empirical-resampling support calibration, not population-sample inference.

**A1 — Supported and aggregation-design-stable**	A5 and A3 did not trigger; the same candidate is resampling-supported at every distinct required vertex of additive $B_{\text{adm}}$.	Strict vertex support extends across the declared domain by frozen linear/common-calibration geometry.	Release that candidate as supported and aggregation-design-stable within $B_{\text{adm}}$.	No stability is claimed outside the declared domain; no universal superiority, future-instance guarantee, deployment validity, or certification follows.

**A2 — Supported but aggregation-design-conditional**	A5 and A3 did not trigger; A1 is false; the reference candidate is supported at *w*_0_; at least one different candidate is resampling-supported at least one distinct required admissible vertex; and a resolved recommendation-changing boundary exists inside $B_{\text{adm}}$.	Different candidates have complete support at distinct required vertices, with a resolved in-domain boundary.	Identify the supported designs or regions and release support as aggregation-design-conditional.	Arbitrary interior support is not an A2 trigger; do not issue one recommendation across the whole admissible domain.

**A4 — Aggregation-design-inconclusive**	A5 and A3 did not trigger; the reference candidate is supported, but neither A1 nor A2 is established after complete frozen design and boundary evaluation.	Reference support is resolved; design status remains unresolved.	State that the reference recommendation is supported but aggregation-design status is unresolved over the declared domain.	Do not convert unresolved geometry into A1, A2, or A5.

### Evidence gates and claim release

The evidence layer distinguishes generic reproducibility/provenance quality from mandatory claim-bearing evidence/authority gates. General provenance and reproducibility maturity may strengthen auditability but does not create simultaneous support, resolve aggregation geometry, or substitute for class-bearing predicates; nonmandatory metadata are not scientific validity gates.

Mandatory claim-bearing requirements are different. They include the authorized evidence identities, portfolio completeness, required denominator/matching records, frozen resampling/design authorities, and class-bearing evidence needed for support and boundary evaluation. A mandatory failure is fail-closed and routes to A5 before stronger recommendation language may be released; it is not recast as ordinary empirical-resampling variability and is not repaired by outcome-dependent changes to the portfolio, coordinate family, weights, threshold, or design domain. Passing the mandatory gates establishes only that the declared decision can be evaluated from authorized evidence. Support and aggregation-design predicates then determine the admissibility state. Thus, evidence governance authorizes the decision path but does not itself supply empirical-resampling support, resolve the boundary, or imply A1.

The released claim is bounded by the declared finite surface, matched portfolio, represented conditions and repeated-evidence basis, empirical-resampling contract, and admissible aggregation-design domain. Recommendation frequencies are descriptive and non-class-determining. The supplementary C6 contribution diagnostics—reference-design group contributions and, where applicable, boundary-movement contributions—are noncausal and non-class-determining. Their formulas and the detailed resampling, coordinate, solver, provenance, and verification records are provided in the Supplementary Material Fig. 1.

**Fig. 1:**
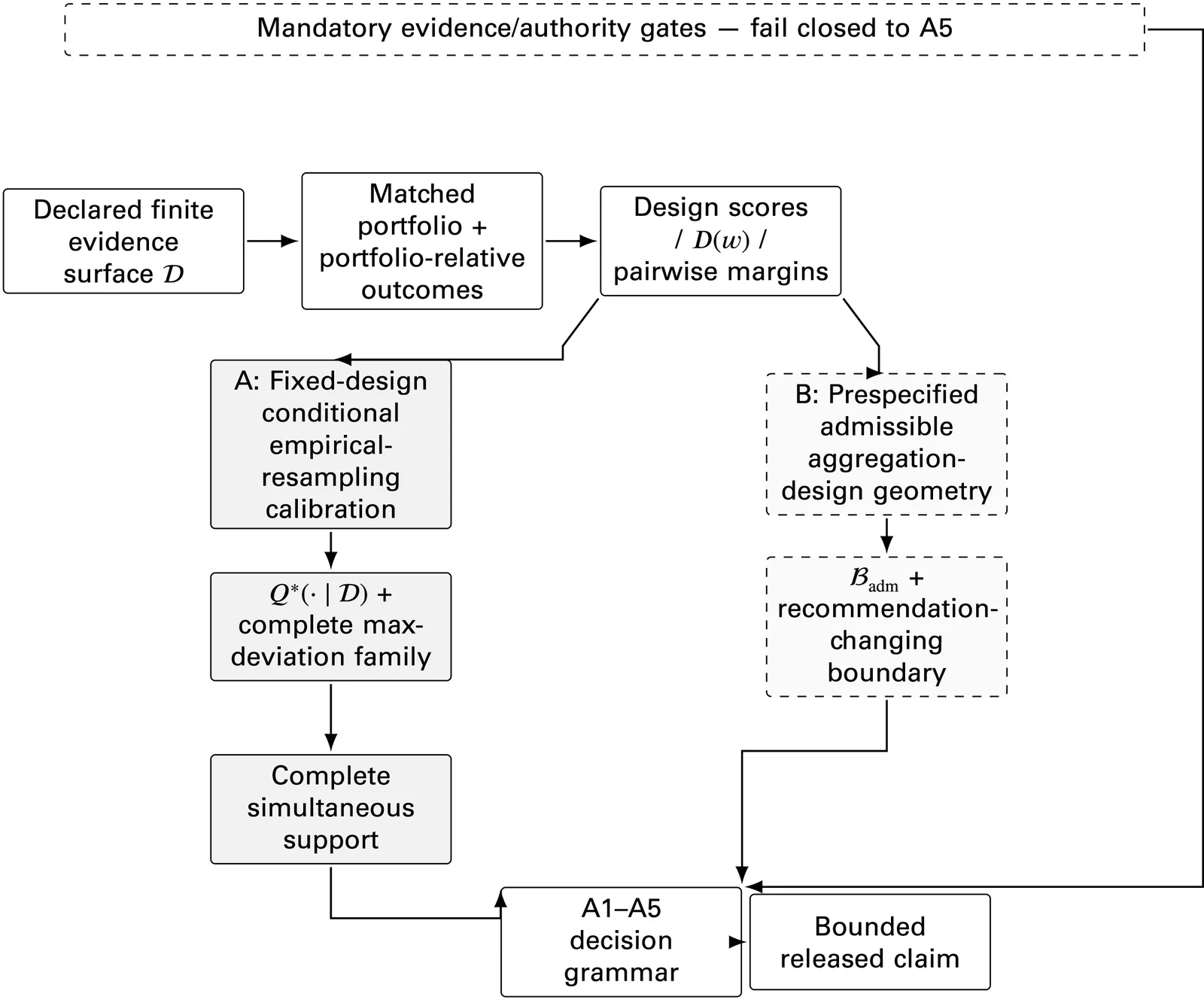
C7 recommendation-admissibility protocol. Empirical resampling and aggregation design remain separate until support and the in-domain boundary enter the A1–A5 grammar. Mandatory evidence/authority gates fail closed to A5; released claims remain bounded to the declared contract.

### Component-method positioning

Computational benchmarking has long treated experimental design and comparison discipline as prerequisites for credible conclusions. Guidance emphasizes matched comparisons, transparent benchmark construction, and qualified interpretation, while algorithm-comparison methods address multiple data sets and repeated evaluations [1], [2], [3], [4]. Bootstrap and related resampling supply general machinery for empirical variation, and benchmark studies show that repeated evaluation conditions can affect comparative conclusions [5], [6]. Multiple-comparison principles likewise matter when one recommendation depends on a family of challenger comparisons: coordinated evidence is needed because uncoordinated pairwise statements do not by themselves support a family-level release. The family-level claim concerns one preferred candidate against the complete declared portfolio, not isolated pairwise findings. C7 uses these established ideas in a claim-bounded way: the declared portfolio is retained under one simultaneous calibration, and a candidate is resampling-supported only when all required simultaneous resampling-calibrated lower bounds against the complete declared portfolio are strictly positive. This is conditional calibration on the represented finite evidence, not a transfer of conventional population-bootstrap semantics or a claim of new resampling or multiplicity mathematics.

A separate benchmark literature shows that winner identity can depend on how heterogeneous evidence is aggregated. Performance profiles and related benchmarking analyses expose aggregation structure rather than reducing all evidence to an uninterpreted average [7], [8], while candidate-election formulations treat benchmark units as contributors to aggregate choice [9]. Vote’n’Rank is the closest benchmark-specific precedent reviewed here because it models systems as candidates and tasks as aggregation units and examines conditions under which a system becomes a winner [10]. Related multicriteria methods—SMAA-2, rank-invariance analysis, inverse weight-sensitivity analysis, and recent weighted-sum stability work—provide established machinery for reasoning about weight uncertainty and ranking stability [11], [12], [13], [14]. C7 draws on this weight-space perspective for its aggregation-design axis, while treating the weights as prespecified scientific target conventions and evaluating that axis separately from finite-evidence support.

An adjacent methodological precedent is the MASINISA framework, which moves from multi-attribute ranking toward rule-based screening and justified choice for industrial robot selection [15]. Its relevance to C7 is the explicit move from ordering alternatives to a governed choice object; the application decision and rule structure are different. Reproducibility and provenance research contributes transparent inputs, inspectable records, and traceable computational workflows that strengthen claim integrity [16], [17], [18]. In C7, these mechanisms authorize the claim-bearing evidence path and enable fail-closed handling when mandatory evidence or authority is unavailable. FJSP provides the empirical domain for demonstrating the integrated decision. Its benchmark tradition supplies heterogeneous instance structures and established comparative settings [19], [20], [21], while scheduling-rule research provides precedent for structured computational comparison of dispatching policies [22], [23]. These characteristics make FJSP suitable for declaring finite evidence surfaces, matched portfolios, represented conditions, repeated-evidence structures, and alternative aggregation conventions.

Prior work therefore supplies each principal component used by C7: matched benchmark comparison and empirical resampling, weight-space sensitivity, and provenance or audit mechanisms. What the literature reviewed here does not supply is a single release rule that requires complete-portfolio conditional resampling support and admissible aggregation-design behavior before a bounded recommendation is issued. C7’s contribution is that integration, not new mathematics for resampling, ranking, weighting, recommendation boundaries, or reproducibility.

## Method validation on two declared finite FJSP evidence surfaces

This section instantiates C7 on two declared finite FJSP evidence surfaces and reports the resulting recommendation-admissibility states. The demonstrations are limited to those surfaces; V2 is not treated as external, population, deployment, or cross-domain validation of V1 or C7.

### FJSP as the empirical evidence domain

Flexible job shop scheduling (FJSP) supplies the empirical domain for instantiating the recommendation-admissibility protocol described in Method details. The role of the domain is deliberately limited. Its structured benchmark collections permit prespecified strata, represented processing-time conditions, repeated-evidence identities, and matched algorithm comparisons without requiring a solver taxonomy or general FJSP performance claim.

Both empirical instantiations use the same exact five-algorithm matched portfolio: ECT, FIFO/ECT, SPT, LPT, and MWKR. Each claim-bearing matched cell retains all five algorithms under the same represented case, processing-time condition, and replication identity, so the portfolio-relative outcome defined in Method details is formed from aligned evidence rather than from algorithm-specific subsets. Three represented processing-time conditions are used on both surfaces: nominal, pt_noise_light, and pt_noise_medium. Replication identities provide the repeated-evidence basis required by the frozen empirical-resampling contracts. They affect the authorized evidence perturbation but do not create additional benchmark target mass.

The finite grouping structure is prespecified before outcome interpretation. V1 preserves benchmark-family and subset identities; V2 uses its fixed work-center  ×  job construction. Processing-time presets are represented conditions rather than new benchmark populations, and replication IDs index repeated evidence under each surface’s frozen coupling. These structures support within-stratum empirical perturbation and declared aggregation targets without a model for unseen FJSP instances.

The two surfaces are separately scoped finite empirical instantiations of the same claim rule. They are not samples from one common statistical population, and neither is used as a numerical benchmark for the other. V1 is the historical/retrospective finite FJSP evidence surface. V2 is the independently and prospectively frozen second finite FJSP evidence surface. Each has its own fixed corpus, grouping structure, admissible aggregation-design geometry, conditional empirical-resampling reference distribution, complete simultaneous coordinate family, and calibration.

### V1: historical/retrospective finite FJSP evidence surface

V1 contains 293 represented benchmark instances from the Brandimarte, DPdata, and Hurink material. The represented cases are partitioned into six fixed family-subset strata: Brandimarte-mk, DPdata-dauzere, and Hurink edata, rdata, sdata, and vdata. Crossing those strata with the three frozen processing-time conditions yields 18 family-subset-preset groups. The primary repeated-evidence basis used for V1 classification is replication IDs 0–9.

The V1 aggregation-design domain expresses three prespecified ways to assign target mass to this represented finite corpus. V_MICRO assigns equal mass per observed instance-preset cell. V_EQUAL_SUBSET assigns equal target mass to each of the six represented family-subset domains and equal mass across its represented presets. V_EQUAL_FAMILY first equalizes the represented benchmark families, then the represented subsets within each family, and then the presets within each subset. The admissible V1 domain is exactly conv{V_MICRO,V_EQUAL_SUBSET,V_EQUAL_FAMILY}.

These vertices are scientific target conventions, not bootstrap outcomes or post-result sensitivity choices. Exact definitions of V_MICRO, V_EQUAL_SUBSET, and V_EQUAL_FAMILY are provided in Supplementary Section S1.11 and Table S5. The declared V1 hull likewise does not admit leave-out constructions, arbitrary group emphasis, or an unrestricted 18-group simplex. For simultaneous calibration, the five-member portfolio contributes 20 ordered algorithm pairs at each of the three distinct vertices, giving a complete frozen family of 60 ordered-pair/design coordinates.

V1 is retrospective: the historical evidence predated reconstruction and freezing of the current analysis contract, and the claim-bearing surface was not reconstructed around later outcomes. The mixed-basis companion analysis is supplementary and does not enter the V1 classification; source authority and provenance are documented in the Supplementary Material.

### V2: independently and prospectively frozen second finite FJSP evidence surface

V2 is the independently and prospectively frozen second finite FJSP evidence surface built from the Behnke-Geiger Work Centers benchmark source. It contains exactly 60 represented static FJSP instances arranged in 12 fixed work-center  ×  job structural strata, with five instances in each stratum. The frozen structural design has three work-center levels and four job levels; crossing the 12 strata with the same three processing-time presets yields 36 structural-stratum-preset groups. Replication IDs 0–9 provide the repeated-evidence basis, and the exact same five-algorithm matched portfolio is retained.

The V2 route, evidence identities, structural/repeated-evidence design, matched execution contract, aggregation-design endpoints, and analysis contract were frozen before outcome-bearing interpretation. Here, ’independently’ refers to the separately frozen V2 evidence route, not to statistical independence or external validation; V2 was not selected because of a later recommendation state. Detailed source, parser, execution, and conformance chronology remains supplementary.

V2 uses two frozen aggregation-design endpoints. V_INSTANCE is the equal-instance target convention for the represented finite corpus. V_OPERATION is an operation-count exposure design and is algebraically identical to job-count weighting on this finite corpus because every job contains exactly five operations. It is not empirical workload, machine load, processing-time exposure, computational difficulty, or industrial importance. The V2 reference design was prospectively frozen before outcome-bearing interpretation as $w_{0}=V\_\mathrm{INSTANCE}$; V_INSTANCE is an approved directly calibrated class-bearing endpoint. Its reference role anchors the V2 A3/reference branch and the recommendation-changing boundary calculation. It does not reduce the A1 requirement: the same candidate must satisfy the frozen support rule at both required endpoints, V_INSTANCE and V_OPERATION. Exact definitions of V_INSTANCE and V_OPERATION are provided in Supplementary Section S1.11 and Table S6. The admissible V2 domain is exactly the closed line segment conv{V_INSTANCE,V_OPERATION}; no additional eligible-assignment endpoint and no unrestricted 36-group simplex is admitted. With 20 ordered algorithm pairs at each of the two endpoints, the complete frozen simultaneous family contains 40 ordered-pair/design coordinates.

### Surface-specific resampling and aggregation-design contracts

The empirical-resampling contracts instantiate the conditional reference law specified in Method details separately on each surface. For V1, the six family-subset domains are fixed strata. Within each stratum, represented instances are drawn with replacement at the frozen stratum draw size, and ten replication IDs are drawn with replacement from 0–9. The selected replication-ID sequence is applied jointly across retained instances, all three represented conditions, and all five matched algorithms according to the frozen V1 coupling rules. Thus the instance stage perturbs the empirical composition of already represented cases and the replication stage perturbs authorized repeated-evidence identities, while matched comparisons are preserved. Nominal replication degeneracy is retained exactly rather than converted into synthetic randomness.

For V2, the 12 work-center  ×  job structural cells are the fixed strata. Five represented instances are drawn with replacement within each stratum, and ten replication IDs are drawn with replacement from 0–9 at the frozen structural-stratum scope. The same selected replication sequence is carried jointly across the retained cases, all represented processing-time conditions, and all five algorithms in that stratum. Pairing and coupling therefore remain aligned across the matched portfolio and represented conditions, including the exact nominal degeneracy.

In both contracts, the strata themselves remain fixed parts of the declared surface. Resampling changes only the multiplicities of represented case identities and authorized replication identities at the scopes just described. It does not resample benchmark families, work-center levels, job levels, presets, algorithms, or aggregation designs as if these were draws from a higher-level population.

Each surface uses 20,000 empirical resamples under the frozen seed 20260802. These common implementation values do not create a shared statistical object. V1 and V2 each induce their own $Q^{*}\left(\cdot |D\right)$ conditional on their own declared finite evidence surface and resampling contract, and each complete simultaneous family is calibrated only within that surface. No critical deviation is transferred from V1 to V2. Throughout both resampling procedures, the aggregation-design target weights remain fixed: empirical bootstrap multiplicities perturb calibration evidence but never redefine target mass, group weights, endpoint weights, or admissible design geometry.

The Method details support semantics apply unchanged. Direct resampling support is calibrated only at the frozen class-bearing fixed designs represented in the simultaneous family: the three V1 vertices and the two V2 endpoints. Interior points of the V1 hull or V2 segment do not create additional bootstrap coordinates or new calibrations. Any later support statement for an interior design can arise only through the additive/common-calibration geometry defined in Method details when the required vertex conditions imply it. Correspondingly, any later classification branch that requires a different supported candidate must obtain that support at a distinct required admissible vertex, not from an arbitrary interior point.

For both surfaces, all mandatory claim-bearing evidence gates passed before the recommendation-admissibility rule was evaluated. This permits application of the declared decision rule but does not itself establish support or determine the final class. Detailed provenance, parser, conformance, execution, and verifier records are provided in the Supplementary Material.

V1 and V2 are calibrated separately and are not numerically comparable; cross-surface interpretation is restricted to the categorical release states reported in Section 3.7.

### V1: deterministic point recommendation without complete support

V1 is the historical/retrospective finite FJSP evidence surface. At each of its three required class-bearing vertices, V_MICRO, V_EQUAL_SUBSET, and V_EQUAL_FAMILY, the frozen point-recommendation map gives D(w)={MWKR}. MWKR therefore remains the point recommendation throughout the declared V1 hull. That result enters the A1–A5 decision rule, but it is not sufficient for a supported release Table 2. The V1 support evaluation retains the complete simultaneous family of 60 ordered-pair/design coordinates. No candidate clears the strict complete-portfolio resampling-support rule at any of the three required vertices, so the support set is empty at all three; MWKR remains point-recommended but is not resampling-supported, and no other candidate is supported. Because the mandatory claim-bearing evidence and authority gates had already passed, this absence of support is a class-bearing empirical result rather than an A5 evidence-authority failure.

**Table 2:** Two finite FJSP evidence-surface contracts. Frozen contracts for the two separately scoped empirical surfaces.

Contract element	V1	V2
**Role / chronology**	historical/retrospective finite FJSP evidence surface. Historical evidence pre-existed reconstruction and freezing of the current analysis contract.	independently and prospectively frozen second finite FJSP evidence surface. Behnke–Geiger route/evidence/design were frozen before outcome-bearing interpretation; *independently* does not mean statistical independence, external/test-set/deployment validation, or a validation hierarchy.

**Finite corpus**	293 represented instances.	60 represented static FJSP instances from the Behnke–Geiger Work Centers source.

**Matched portfolio**	ECT; FIFO/ECT; SPT; LPT; MWKR in every authorized matched cell.	ECT; FIFO/ECT; SPT; LPT; MWKR in every authorized matched cell.

**Conditions / repeated-evidence basis**	nominal, pt_noise_light, pt_noise_medium; replication IDs 0–9.	nominal, pt_noise_light, pt_noise_medium; replication IDs 0–9.

**Grouping / strata**	Six fixed family-subset strata: Brandimarte-mk, DPdata-dauzere, Hurink edata/rdata/sdata/vdata; 18 groups.	12 fixed work-center × job strata, five instances each, from 3 work-center × 4 job levels; 36 groups.

**Admissible design domain**	conv{V_MICRO, V_EQUAL_SUBSET, V_EQUAL_FAMILY}. V_MICRO: equal mass per observed instance-preset cell. V_EQUAL_SUBSET: equal target mass to each of six family-subset domains and across its presets. V_EQUAL_FAMILY: equalize families, then represented subsets within family, then presets within subset.	conv{V_INSTANCE, V_OPERATION}. V_INSTANCE: equal-instance target. V_OPERATION: operation-count exposure, algebraically identical to job-count weighting because every job has five operations; not workload, machine load, processing-time exposure, difficulty, or industrial importance. No eligible-assignment endpoint or unrestricted simplex.

**Empirical-resampling contract**	Within each of six fixed strata, sample represented instances with replacement at the frozen stratum size and ten IDs with replacement from 0–9; apply the selected sequence jointly across retained instances, all conditions, and all algorithms. Target weights fixed; nominal degeneracy retained. 20,000 resamples; seed 20260802; surface-specific $Q^{*}\left(\cdot |D\right)$.	Within each of 12 fixed strata, sample five instances with replacement and ten IDs with replacement from 0–9 at structural-stratum scope; carry the same sequence jointly over retained cases, all conditions, and all algorithms. Target weights fixed; nominal degeneracy retained. 20,000 resamples; seed 20260802; surface-specific $Q^{*}\left(\cdot |D\right)$.

**Complete simultaneous family**	20 ordered pairs × 3 frozen vertices = 60 coordinates; direct support only at those vertices before permitted geometric extension.	20 ordered pairs × 2 frozen endpoints = 40 coordinates; direct support only at those endpoints before permitted geometric extension.

**Evidence-gate status**	Mandatory claim-bearing gates passed for evaluation only; detailed provenance/audit evidence is supplementary.	Mandatory prospective source/design/ execution/ conformance and claim-bearing gates passed for evaluation only; detailed chronology/audit evidence is supplementary.

The released V1 state is therefore A3 — Sampling-inconclusive: under the frozen empirical-resampling calibration, no candidate satisfies the complete simultaneous support predicate at the required class-bearing vertices. MWKR nevertheless remains the deterministic point recommendation under the frozen portfolio-relative scoring rule. The admissible V1 claim is therefore point-level only; A3 neither establishes algorithm equivalence nor supports an alternative algorithm.

The V1 recommendation-changing boundary result is BOUNDARY_OUTSIDE_B_ADM: no recommendation-changing tie occurs within the declared V1 aggregation-design domain, consistent with MWKR remaining the point recommendation over the hull. This is a design-geometry result only. Because complete resampling support remains absent, the boundary result does not alter the A3 classification.

### V2: bounded supported recommendation across the declared segment

V2 is the independently and prospectively frozen second finite FJSP evidence surface. Its required class-bearing designs are the two endpoints V_INSTANCE and V_OPERATION, and at both endpoints D(w)={ECT}. ECT is thus the deterministic point recommendation at both required endpoints, after which the frozen support rule determines whether stronger recommendation language is admissible.

The V2 support evaluation retains the complete 40-coordinate ordered-pair/design family. ECT is resampling-supported at both required endpoints: each of the four ECT-versus-challenger simultaneous resampling-calibrated lower bounds is strictly positive at V_INSTANCE and at V_OPERATION, so all 8/8 ECT-relevant endpoint requirements are strictly positive. These are candidate-relevant consequences of the complete prespecified family, which also retains the authorized reverse-pair and non-ECT coordinates. The mandatory claim-bearing gates had already passed, so the support result—not evidence authorization—determines the subsequent classification.

Direct support is calibrated only at V_INSTANCE and V_OPERATION. Because the relevant margins are linear in the aggregation weights and use the same calibrated subtraction, strict positivity at both endpoints implies strict positivity throughout their closed segment; no interior resampling or recalibration is performed. ECT therefore receives A1 — Supported and aggregation-design-stable within the declared V2 surface, portfolio, conditions, replication basis, and aggregation-design segment. BOUNDARY_OUTSIDE_B_ADM additionally establishes that no recommendation-changing tie occurs inside that segment, but it is not the basis for the A1 classification.

### Categorical interpretation across the two finite surfaces

The two empirical surfaces remain separate finite instantiations, each with its own $Q^{*}\left(\cdot |D\right)$, coordinate family, and critical calibration. Their normalized regrets, calibration values, empirical-resampling variability, margins or lower bounds, and boundary distances are not numerically compared because those quantities arise under different finite targets and surface-specific calibrations. The admissible comparison is only between the realized categorical state sequences stated above. V1 shows that a resolved point recommendation can remain unreleased as a supported recommendation when complete support is absent; V2 shows that bounded support can be released when the frozen endpoint and aggregation-design predicates are jointly satisfied. A1 and A3 are claim-release states, not performance tiers. V2 does not validate V1 or C7, and no uncertainty ordering is asserted. The different recommended algorithms likewise do not imply an ECT-versus-MWKR performance ordering Table 3.

**Table 3:** Released recommendation-admissibility outcomes. Class-bearing outcomes for the two separately scoped finite FJSP evidence surfaces under the common frozen protocol.

Result element	V1	V2
**Point recommendation**	Historical/retrospective finite FJSP evidence surface: D(w)={MWKR} at all three required vertices; unchanged over the declared V1 design hull.	Independently and prospectively frozen second finite FJSP evidence surface: D(w)={ECT} at both required endpoints.

**Complete simultaneous support**	Complete 60-coordinate family retained; no candidate clears the strict complete-portfolio resampling-support rule; support set empty at all three vertices.	Complete 40-coordinate family retained; ECT resampling-supported at both endpoints; four challenger requirements positive per endpoint, i.e. 8/8 ECT-relevant requirements strictly positive.

**Aggregation-design / boundary result**	BOUNDARY_OUTSIDE_B_ADM; this point-geometry result does not create support.	Endpoint positivity plus the frozen additive linear-margin/common-calibration geometry carries ECT support through the closed declared segment; no interior recalibration is introduced. BOUNDARY_OUTSIDE_B_ADM.

**Released recommendation-admissibility class**	A3 — Sampling-inconclusive.	A1 — Supported and aggregation-design-stable.

**Bounded scientific interpretation**	MWKR is point-only; the surface is inconclusive under the frozen empirical-resampling support calibration.	ECT is a scope-bounded supported recommendation only within the declared V2 finite surface, portfolio, conditions, replication basis, and aggregation-design segment.

**Cross-surface restriction**	Categorical sequence only: point recommendation → no complete support → A3; no numerical cross-surface comparison.	Categorical sequence only: point recommendation → endpoint complete support → permitted segment extension → A1; A1/A3 are claim-release states, not performance tiers or validation.

*Table note.* The surfaces have separate conditional empirical-resampling reference laws and calibrations. Numerical regret, calibration, empirical-resampling variability, margin/lower-bound, and boundary-distance magnitudes are not cross-surface comparable.

## Limitations

**Finite target and portfolio.** Claims concern only the represented finite evidence surfaces, their frozen conditions, and the declared five-algorithm portfolio. They do not support unseen-instance, superpopulation, iid all-problems, manufacturing-population, or design-based finite-population inference. Normalized regret is portfolio-relative, not an exact-optimum or oracle gap.

**Calibration and support scope.** The resampling law is conditional on frozen represented evidence and the authorized perturbation contract. The 0.95 level is a nominal empirical-resampling decision calibration, not confidence coverage, a truth probability, or a probability of future success. Direct support is evaluated only at frozen class-bearing designs through the complete simultaneous family and strict lower-bound predicate; permitted interior support follows only from the frozen additive linear-margin/common-calibration geometry, not a new bootstrap coordinate or calibration.

**Aggregation and release scope.** Claims extend only over the declared $B_{\text{adm}}$. BOUNDARY_OUTSIDE_B_ADM does not imply stability over an unrestricted simplex or all plausible preferences. Only A3 and A1 are empirically realized; A2, A4, and A5 remain protocol-defined states. A1 and A3 are release states, not performance tiers.

**Chronology, comparison, and reproducibility.** V2 being independently and prospectively frozen does not imply statistical independence, external, test-set, deployment, industrial, or other validation of V1 or C7. V1 and V2 have separate targets, coordinate families, and calibrations; regret, calibration, empirical-resampling variability, margins, lower bounds, and boundary distances are not numerically compared. C6 and recommendation-frequency outputs are diagnostic, noncausal, and non-class-determining. Provenance supports bounded auditability, not support or certification; complete end-to-end regeneration from every upstream historical experimental origin is not claimed. These limits preclude universal FJSP, deployment, certification, and future-instance claims.

## Ethics approval

Not applicable.

## Consent to participate

Not applicable.

## Consent for publication

Not applicable.

## Declaration of generative AI and AI-assisted technologies in the manuscript preparation process

During manuscript preparation, the author used OpenAI ChatGPT (GPT-5.4) to assist with language editing, stylistic refinement, and the drafting of manuscript text. All AI-assisted content was critically reviewed, revised where necessary, and approved by the author. The author takes full responsibility for the accuracy, integrity, interpretation, and final content of the manuscript.

## Declaration of competing interest

The author has no relevant financial or non-financial interests to disclose.

## Data Availability

The journal Supplementary Material is a separate reader-facing S1–S6 scientific record. Supporting evidence, code, schemas, contracts, derived artifacts, provenance, and integrity material are available in the public Figshare evidence-support package [24] at https://doi.org/10.6084/m9.figshare.33287469. Raw third-party benchmark source files are not redistributed. Complete end-to-end regeneration from every upstream historical experimental origin is not claimed.
