# Supplementary material for: Recommendation admissibility for finite computational benchmarks under empirical-resampling variability and aggregation-design uncertainty
Source: MethodsX. 2026 Nov 2;17:104238. doi: 10.1016/j.mex.2026.104238 (PMC13756293; doi:10.1016/j.mex.2026.104238)
Supplement: Supplementary file 1 [file mmc1.pdf]

Supplementary Material: Recommendation Admissibility for Finite Computational Benchmarks under Empirical-Resampling Variability and Aggregation-Design Uncertainty

This document contains Supplementary Material S1–S6 for this article. It presents the protocol, exact aggregation-design definitions, evidence records, coordinate tables, diagnostics, and scientific provenance needed to interpret the manuscript independently. The supplement neither recomputes scientific results nor enlarges any finite-evidence-surface, portfolio, uncertainty, or aggregation-design scope.

1. S1 — Complete protocol and analysis contracts.
2. S2 — V1 evidence, calibration, and bounded diagnostics.
3. S3 — V2 source, execution, and conformance provenance.
4. S4 — Complete simultaneous-coordinate evidence.
5. S5 — Diagnostic and noncausal contribution material.
6. S6 — Claim interpretation, scientific provenance, and reproducibility boundary.

Reproducibility boundary. Supporting evidence records preserve the frozen protocol, V1/V2 evidence, provenance, and integrity checks. Raw third-party benchmark source files are not redistributed. Complete end-to-end regeneration of every upstream historical experimental origin is not claimed.

S1. Complete Protocol and Analysis Contracts

S1.1 Scope and interpretation notice

This supplement documents the frozen recommendation-admissibility protocol and the two surface-specific analysis contracts used by the manuscript. It does not introduce a method, modify an estimand, add a benchmark surface, or alter an empirical recommendation, support set, boundary outcome, critical value, or A1–A5 assignment. Its purpose is to make the predeclared objects, gates, and interpretation limits readable without repeating the main manuscript verbatim.

The protocol addresses the admissibility of a recommendation derived from a declared finite benchmark evidence surface. It does not certify a scheduling rule, establish deployment validity, or convert the represented benchmark cases into a random sample of all flexible job shop problems or manufacturing systems. Its reproducibility statement is bounded: the frozen evidence record is inspectable, but complete end-to-end regeneration from every upstream historical experimental origin is not claimed.

S1.2 Finite evidence surface and matched portfolio

Let a finite evidence surface contain benchmark cases partitioned into groups $\mathcal{G}$, fixed uncertainty conditions, an authorized repeated-evidence structure, and a finite matched portfolio $\mathcal{A}$. An authorized outcome row identifies a case, condition, repeated-evidence identity where applicable, and one member of $\mathcal{A}$. A matched cell c is the collection of authorized rows for every portfolio member under the same case, condition, and repeated-evidence identity. Claim-bearing cells are complete: no portfolio member may be absent, imputed, or assigned an algorithm-specific denominator.

For the lower-is-better outcome $y_{c,a}$ of algorithm $a \in \mathcal{A}$, the frozen cell-level quantity is portfolio-relative normalized regret,

$$r_{c,a} = \frac{y_{c,a} - \min_{b \in \mathcal{A}} y_{c,b}}{\min_{b \in \mathcal{A}} y_{c,b}}. \tag{1}$$

Every member attaining the within-cell minimum, including a tied minimum, receives zero regret. The denominator must be positive. A nonpositive denominator excludes the entire matched cell for every portfolio member, records the exclusion, and invokes the applicable evidence gate; a missing member cannot be repaired by silent imputation or renormalization. Equation (1) is relative to the best observed member of the declared portfolio in the same cell. It is not an optimality gap to an oracle, exact solver, external lower bound, or certified optimum.

Every surface contract explicitly fixes its within-group operator before outcome interpretation; it cannot remain unspecified or be selected after results are observed. For the two surfaces reported here, $R_{g,a}$ is the arithmetic mean of all authorized matched cells in group g after the whole-cell exclusion rule, using the same authorized cell basis for every algorithm. No whole-cell exclusion was exercised because every authorized within-cell minimum was positive. For V1 group $g = (s, p)$, with represented-instance set I_s, $n_s = |I_s|$, preset p, and replication ID h,

$$R_{(s,p),a} = \frac{1}{10 n_s} \sum_{i \in I_s} \sum_{h=0}^{9} r_{(i,p,h),a}. \tag{2}$$

For V2 structural stratum s and preset p,

$$\bar{R}_{i,p,a} = \frac{1}{10} \sum_{h=0}^{9} r_{(i,p,h),a}, \qquad R_{(s,p),a} = \frac{1}{5} \sum_{i \in I_s} \bar{R}_{i,p,a}. \tag{3}$$

Thus the V2 group operator is also the arithmetic mean over its $5 \times 10 = 50$ authorized matched cells. With a declared nonnegative group-weight vector w satisfying $\sum_{g \in \mathcal{G}} w_g = 1$, the design score and set-valued point recommendation are

$$\theta_a(w) = \sum_{g \in \mathcal{G}} w_g R_{g,a}, \qquad D(w) = \arg\min_{a \in \mathcal{A}} \theta_a(w). \tag{4}$$

The recommendation is deliberately set-valued. A lexical, file-order, or display tie-break may not create a claim-bearing unique recommendation. The reference design w_0 is an explicit declared benchmark target convention, not a normative truth or universally preferred weighting. It belongs to $\mathcal{B}_{\mathrm{adm}}$ and is a prespecified directly calibrated class-bearing design included in the simultaneous coordinate family.

This construction fixes two otherwise easily conflated objects. The first is a cell-level comparison: the matched portfolio determines the within-cell minimum in Equation (1). The second is an aggregation-design target convention: the group-weight vector determines how the already constructed group quantities enter a design score. Neither object is inferred from a preferred point result. A different algorithm portfolio changes

the comparator and requires a new surface; a different declared benchmark target convention is admissible only when it belongs to the prospectively declared design set.

The completeness gate has the same purpose. It does not attempt to rescue an incomplete comparison by discarding one algorithm or by averaging its available rows over a different denominator. Instead, the matched cell is the smallest claim-bearing unit. This preserves the meaning of a portfolio-relative comparison under both the fixed-design score and the resampling contract. Any exclusion must be surface-wide, recorded with its reason and affected identities, and evaluated by the classification logic rather than hidden in a summary statistic.

S1.3 Separate uncertainty axes

Empirical-resampling variability concerns the stability of estimated group quantities, pairwise margins, recommendation identity, and related quantities when an aggregation design is held fixed. Each surface addresses it through its own frozen dependence-preserving resampling contract. Aggregation-design uncertainty is different: it concerns movement among prospectively specified declared benchmark target conventions. It is assessed over the declared admissible aggregation-design set $\mathcal{B}_{\mathrm{adm}}$.

The latter set is not a probability distribution, an empirical bootstrap resample of weights, or an unrestricted simplex unless that interpretation has independently been justified and frozen. Its support, vertices, mass constraints, and scientific meanings are part of the estimand. Resampling multiplicities describe an empirical bootstrap resample; they never revise declared benchmark target weights. The protocol combines the two axes only through its decision rules: support is assessed at fixed designs, and support status is then examined over the surface's own declared design domain.

The distinction also prevents a sensitivity display from being mistaken for inference. A design vertex says what declared benchmark target convention is being represented; it is not a random alternative outcome. Conversely, a bootstrap replicate says how the authorized empirical units are resampled under one contract; it is not a newly admissible benchmark target. The protocol therefore reports resampling support and design geometry side by side, but does not average the two into one probability, rank, or unrestricted robustness score. A declared domain may be small when only a small number of target conventions have scientific interpretation. That restriction is a feature of claim discipline, not an invitation to search post hoc for a more favorable weighting.

S1.4 Complete-portfolio simultaneous support

For lower-is-better design scores, the ordered pairwise margin is

$$M_{a,b}(w) = \theta_b(w) - \theta_a(w) = \sum_{g\in\mathcal{G}} w_g\big(R_{g,b} - R_{g,a}\big). \tag{5}$$

Thus $M_{a,b}(w) > 0$ favors a over b, zero is their tie boundary, and a negative value favors b. Conditional on the frozen evidence surface $\mathcal{D}$, the paired stratified two-way empirical-resampling bootstrap induces the conditional empirical-resampling reference distribution $Q^*(\cdot \mid \mathcal{D})$ over authorized perturbations. The surface-specific procedure constructs the complete declared family of ordered margins and simultaneous resampling-calibrated lower bounds $B_{a,b}(w)$. A candidate is resampling-supported at w only when

$$B_{a,b}(w) > 0 \qquad \text{for every } b \in \mathcal{A}\setminus\{a\}. \tag{6}$$

This is a strict complete-portfolio requirement. A point ranking, a marginal interval, a selected comparison, a conditional empirical-resampling recommendation frequency, or an unadjusted pairwise analysis cannot replace Equation (6). Conditional empirical-resampling recommendation frequencies are descriptive summaries, not support thresholds or probabilities that a rule is generally best.

The simultaneous family is declared before the result is interpreted. It includes every ordered pair and every distinct fixed design required by the relevant surface contract, including the prespecified directly calibrated class-bearing reference design w_0, even when a later point calculation makes some comparisons appear less consequential. Keeping the family complete prevents outcome-driven narrowing to a convenient challenger or a candidate-only calibration. The common lower-bound rule also means that the displayed candidate must clear every retained challenger under the same familywise calibration. It does not mean that an algorithm has been compared with every possible solver outside the declared portfolio.

For a surface with complete coordinate family $k = 1,\ldots,K$, let M_k be its point ordered-pairwise margin and $M^*_{r,k}$ the corresponding margin in empirical bootstrap resample r. The frozen unstudentized directional max-deviation construction is

$$\delta_{r,k} = M_k - M^*_{r,k}, \qquad z_r = \max_{1\le k\le K} \delta_{r,k}. \tag{7}$$

With the frozen linear empirical 0.95-quantile rule $q_{0.95}^{\text{linear}}$, the surface-specific critical deviation and simultaneous resampling-calibrated lower bound are

$$c_{0.95} = q_{0.95}^{\text{linear}}\{z_1, \ldots, z_R\}, \qquad B_k = M_k - c_{0.95}, \qquad R = 20{,}000. \tag{8}$$

For sorted values $z_{(1)} \le \cdots \le z_{(R)}$, the frozen NumPy `quantile(..., method="linear")` convention uses $h = (R-1)p$, $j = \lfloor h \rfloor$, and $\gamma = h - j$, where j is zero-based:

$$q_p^{\text{linear}} = (1-\gamma)z_{(j+1)} + \gamma z_{(j+2)}. \tag{9}$$

At $R = 20{,}000$ and $p = 0.95$, $h = 18{,}999.05$ and

$$c_{0.95} = z_{(19000)} + 0.05\big[z_{(19001)} - z_{(19000)}\big]. \tag{10}$$

The value 0.95 is the prespecified nominal empirical-resampling decision-calibration level of this frozen reference procedure, not frequentist coverage for an unobserved parameter. The implementation remains the complete ordered directional family: at every required fixed design both (a, b) and (b, a) are retained, with $M_{b,a} = -M_{a,b}$, $M^*_{r,b,a} = -M^*_{r,a,b}$, and therefore $\delta_{r,b,a} = -\delta_{r,a,b}$. Consequently, the implemented maximum over all ordered directional coordinates is algebraically equal to the maximum of $|\delta_{r,k}|$ over one representative of each unordered pair/design. This equivalence is a consequence of retaining every reverse coordinate; it does not replace the frozen implementation $\delta_{r,k} = M_k - M^*_{r,k}$, $z_r = \max_k \delta_{r,k}$, and it changes no frozen computation, coordinate evidence, calibration, or support result. The construction is not replaced by percentile intervals, studentized intervals, Bonferroni intervals, or an algorithm-wise family. Each surface has its own family and calibration record; the shared resampling seed does not create a common inferential scale or permit a calibration to be transferred from one surface to the other.

For V1 resample r, let $I_{r,s,j}$ and $H_{r,s,\ell}$ denote the resampled instance and replication identities in stratum s. Then

$$R^*_{r,(s,p),a} = \frac{1}{10 n_s} \sum_{j=1}^{n_s} \sum_{\ell=1}^{10} r_{(I_{r,s,j}, p, H_{r,s,\ell}), a}. \tag{11}$$

For V2,

$$R^*_{r,(s,p),a} = \frac{1}{50} \sum_{j=1}^{5} \sum_{\ell=1}^{10} r_{(I_{r,s,j}, p, H_{r,s,\ell}), a}. \tag{12}$$

Duplicate instance identities and duplicate replication IDs contribute according to their sampled multiplicities. Within each frozen stratum, the same draw vectors are applied jointly across represented presets and all five algorithms, preserving matched coupling; bootstrap multiplicities do not alter aggregation target weights.

S1.5 Boundary contract

Boundary reporting begins only if $D(w_0) = \{a_0\}$ is unique. Every retained challenger is then searched within the complete portfolio. Under the frozen distance d, the nearest admissible recommendation-changing design is

$$\rho = \min_{b \ne a_0} \; \min_{\substack{w \in \mathcal{B}_{\text{adm}}: \\ M_{a_0,b}(w) = 0}} d(w, w_0). \tag{13}$$

For probability-weight designs, the frozen total-variation convention is

$$TV(w, w_0) = \frac{1}{2}\|w - w_0\|_1. \tag{14}$$

It represents benchmark mass reallocated across groups; it is not a distance in an unrestricted weight space.

An inside boundary is a feasible tie in $\mathcal{B}_{\text{adm}}$ and carries its active challenger set, distance, and constraint record. An outside boundary means that no challenger tie intersects the declared domain; its distance is null or undefined, not substituted with an unrestricted-domain value. An unresolved boundary records incomplete decision-critical geometry under an otherwise authorized surface. Multiple active challengers are retained together. If a nearest design is nonunique, its optimal face is reported through ranges rather than an arbitrary returned point. These are established boundary-reporting tools used under a prespecified domain; they are not claimed as new mathematics.

Boundary reporting is deliberately subordinate to support. It describes where a unique point recommendation would change under the declared aggregation geometry; it cannot supply simultaneous evidence that is absent at a fixed design. Nor may an outside boundary be restated as universal stability. Its appropriate interpretation is entirely conditional on the declared finite evidence surface, matched portfolio, within-group aggregation, vertices, and constraints defining the particular $\mathcal{B}_{\text{adm}}$. The evidence record retains the active-set, feasibility, tolerance, and null-distance fields so that a reader can distinguish a genuine in-domain boundary from a lack of a permitted boundary calculation.

S1.6 A1–A5 classification logic

The primary class is evaluated in exactly this frozen precedence order:

$$\texttt{A5} \rightarrow \texttt{A3} \rightarrow \texttt{A1} \rightarrow \texttt{A2} \rightarrow \texttt{A4}.$$

The order first separates absent or unauthorized evidence from valid uncertainty, then prevents a point result from being promoted without simultaneous support. A3 and A4 are unresolved evidence states, not lower-quality locations on a performance scale. Table S1 gives the complete classification grammar.

The classification logic is intentionally claim-bounded rather than winner-bounded. It does not ask merely whether one portfolio member has the smallest reported score. It asks whether the required evidence is present, whether the reference-design recommendation is resolved under the complete simultaneous family, and whether the relevant supported status persists over the declared aggregation-design domain. This makes the class record informative even when it withholds a stronger recommendation statement.

Table S1: S1 classification precedence and interpretation.

Class	Trigger and implication
A5	A mandatory claim-bearing evidence or authority requirement is missing, unauthorized, inconsistent, or invalid; no substantive recommendation-admissibility claim is released.
A3	A5 has not triggered and the reference recommendation is tied, no candidate clears complete resampling support at the prespecified directly calibrated class-bearing w_0, or a decision-critical reference margin remains unresolved; the result is sampling-inconclusive.
A1	A5 and A3 have not triggered and the same candidate is resampling-supported at every distinct required admissible vertex or endpoint of the declared additive aggregation-design domain.
A2	A5 and A3 have not triggered; A1 is false; the reference candidate is supported at w_0; at least one different candidate is resampling-supported at least one distinct required admissible vertex or endpoint; and a resolved recommendation-changing boundary exists inside the declared admissible aggregation-design domain.
A4	A5 and A3 have not triggered; the reference candidate is supported, but neither A1 nor A2 is established after complete frozen aggregation-design and boundary evaluation; the aggregation-design status remains unresolved.

Table S2: V1 frozen contract.

Field	Contract
Surface	Historical/retrospective finite FJSP surface; 293 instances, 18 groups, five-rule matched portfolio.
Designs	$\mathrm{conv}\{V_{\mathrm{MICRO}}, V_{\mathrm{EQUAL\_SUBSET}}, V_{\mathrm{EQUAL\_FAMILY}}\}$; exact definitions in S1.11.
Resampling	Paired stratified two-way empirical resampling on replication identifiers 0–9; 20,000 resamples and 60 ordered coordinates.
Interpretation	MWKR point-only; empty required support; A3 is sampling-inconclusive; `BOUNDARY_OUTSIDE_B_ADM`.

S1.7 V1 contract

V1 is the historical/retrospective finite FJSP evidence surface. Its confirmatory contract uses the common uniform replication-ID basis 0–9. A historical mixed-basis companion is retained for descriptive context only; it is not substituted into the class-determining simultaneous inference. The V1 design domain is a constrained three-vertex hull, and its empirical-resampling contract preserves paired instance and replication dependence without allowing resampling multiplicities to become benchmark weights. Table S2 records the compact V1 contract together with its frozen bounded interpretation; detailed empirical evidence is reported later in this Supplement.

The historical/retrospective role matters for provenance rather than for a quality ranking. The uniform primary basis prevents targeted replication depth from changing benchmark mass in the confirmatory estimand. The companion basis is descriptive and nonconfirmatory; it cannot alter the V1 reference design, the 60-coordinate family, or the class precedence.

Table S3: V2 frozen contract.

Field	Contract
Surface	Independently and prospectively frozen finite FJSP surface; 60 instances, 36 groups, five-rule matched portfolio.
Designs	$\mathrm{conv}\{V_{\text{INSTANCE}}, V_{\text{OPERATION}}\}$; exact definitions in S1.11.
Reference design	$w_0 = V_{\text{INSTANCE}}$, prospectively fixed before outcome-bearing interpretation and directly calibrated.
Resampling and A1	20,000 paired stratified two-way resamples and 40 ordered coordinates; the same candidate must be supported at both endpoints.
Interpretation	ECT supported at both endpoints; eight ECT-relevant endpoint requirements positive; A1; `BOUNDARY_OUTSIDE_B_ADM`.

Table S4: Permitted interpretation boundaries.

Item	Permitted interpretation
Support	Strictly positive simultaneous lower bounds against every challenger in the declared portfolio.
Boundary	Conditional geometry within the declared admissible design domain, not universal stability.
Calibration	Prespecified conditional empirical-resampling decision calibration, not population coverage.
Diagnostics	C6 is diagnostic, noncausal, and non-class-determining.
Scope	Finite evidence-surface statement only; no solver certification, deployment claim, or universal FJSP conclusion.

S1.8 V2 contract

V2 is the independently and prospectively frozen second finite FJSP evidence surface, based on Behnke–Geiger, with its own balanced structural design, matched-cell construction, and two-vertex aggregation-design segment. The prospective source, execution, conformance, and evidence gates are part of the V2 contract before inferential interpretation. Its paired stratified two-way empirical-resampling bootstrap preserves joint instance, replication, preset, and algorithm alignment inside fixed structural strata. Table S3 records the contract and its result interpretation.

The V2 contract keeps design factors and inferential roles distinct. Structural strata define the declared finite evidence surface; presets are represented fixed conditions; replication IDs define repeated uncertainty identities; and the matched portfolio fixes the within-cell comparator. The two aggregation vertices operate only after frozen within-group aggregation. They are aggregation-design target conventions, not uncertainty scenarios or adversarial stress tests. The resampling contract preserves these relationships by drawing the authorized empirical units jointly within the locked strata, while the design weights remain frozen. The prospective lock sequence ensures that these definitions precede quality-bearing analysis; it does not extend the result beyond the represented finite surface.

S1.9 Fail-closed handling and diagnostic exclusions

Mandatory evidence failures are fail-closed. Missing or unauthorized inputs, incomplete matched cells, an invalid required denominator record, a missing required coordinate, an inconsistent design specification, or a failed required validation route the classification to A5 rather than being silently dropped or repaired after inspection. A rendering defect requires targeted implementation repair and revalidation; it is not itself scientific evidence about a portfolio member.

Not every unavailable operational field is a mandatory scientific gate. Where a contract records resource telemetry as unavailable, it must remain 'UNAVAILABLE'; it may not be restated as measured zero. Conversely, absence of a required identity, outcome, schedule-conformance, or inference field cannot be treated as harmless telemetry.

Point and conditional empirical-resampling recommendation frequencies remain descriptive. C6 contribution quantities decompose an explicitly declared margin and, where an inside boundary exists, a declared weight movement. They are diagnostic, noncausal, and non-class-determining. They may not supply a mechanism, a support threshold, or a second route to a class assignment.

The same distinction governs provenance and integrity checks. They confirm the identity and consistency

of retained records; they do not establish that any scheduling rule is universally preferable. Explicit exclusions, including raw artifacts that are not distributed, are stated so that an absent artifact is not mistaken for a complete rerun path.

S1.10 Permitted and prohibited interpretation matrix

Table S4 gives the compact interpretation boundary.

S1.11 Exact aggregation-design definitions

The following definitions are the complete aggregation-design specification for the two finite evidence surfaces. They define group weights, not bootstrap multiplicities. All quantities are exact rational numbers.

V1

Let s denote one of the six family–subset strata and let p denote one of the three processing-time presets. The stratum sizes and the resulting per-group weights are shown in Table S5. Write n_s for the number of represented instances in stratum s, q_s for the frozen V_{MICRO} micro-weight numerator of one preset group in that stratum, and h_f for the number of represented subsets in family f. In this V1 contract $q_s = n_s$; across all three presets, $\sum_{s,p} q_s = 879$, while $\sum_s n_s = 293$. For group $g = (s, p)$,

$$V_{\text{MICRO},g} = \frac{q_s}{879}, \tag{15}$$

$$V_{\text{EQUAL\_SUBSET},g} = \frac{1}{6 \cdot 3} = \frac{1}{18}, \tag{16}$$

$$V_{\text{EQUAL\_FAMILY},g} = \frac{1}{3\, h_f\, 3} = \frac{1}{9h_f}. \tag{17}$$

Thus V_{MICRO} gives equal mass to every instance–preset cell, $V_{\text{EQUAL\_SUBSET}}$ gives equal mass to every family–subset and then to its presets, and $V_{\text{EQUAL\_FAMILY}}$ first gives equal mass to the three families, then to their subsets, and then to presets. The admissible V1 set is exactly

$$\mathcal{B}^{(V1)}_{\text{adm}} = \operatorname{conv}\{V_{\text{MICRO}}, V_{\text{EQUAL\_SUBSET}}, V_{\text{EQUAL\_FAMILY}}\} = \left\{ \sum_v \lambda_v v : \lambda_v \geq 0,\ \sum_v \lambda_v = 1 \right\}. \tag{18}$$

No leave-out construction, additional vertex, arbitrary group emphasis, or unrestricted 18-group simplex belongs to this set.

Table S5: Exact V1 structural quantities and per-group weights. Each displayed weight applies separately to each of the three presets; q_s is the frozen micro-weight numerator and h_f is the number of subsets in the family.

Family	Subset	n_s	q_s	h_f	V_{MICRO}	$V_{\text{EQUAL\_SUBSET}}$	$V_{\text{EQUAL\_FAMILY}}$
Brandimarte	mk	15	15	1	5/293	1/18	1/9
DPdata	dauzere	18	18	1	6/293	1/18	1/9
Hurink	edata	65	65	4	65/879	1/18	1/36
Hurink	rdata	65	65	4	65/879	1/18	1/36
Hurink	sdata	65	65	4	65/879	1/18	1/36
Hurink	vdata	65	65	4	65/879	1/18	1/36

V2

Let $m \in \{5, 10, 15\}$ be the work-center level, $j \in \{10, 20, 50, 100\}$ the job level, and p one of the three presets. Every structural cell (m, j) contains five instances and every job has five operations. For each group $g = (m, j, p)$,

$$V_{\text{INSTANCE},g} = \frac{1}{36}, \tag{19}$$

$$V_{\text{OPERATION},g} = \frac{5 \cdot j \cdot 5}{40500} = \frac{j}{1620}. \tag{20}$$

The total denominator is $40500 = 3 \times 3 \times 5 \times 5 \times (10 + 20 + 50 + 100)$ operations across structural cells and presets. Table S6 gives the four exact weight values, each repeated across the three work-center levels and three presets. The reference design is prospectively fixed as $w_0 = V_{\text{INSTANCE}}$, and

$$\mathcal{B}_{\text{adm}}^{(V2)} = \text{conv}\{V_{\text{INSTANCE}}, V_{\text{OPERATION}}\} = \{(1-\lambda)V_{\text{INSTANCE}} + \lambda V_{\text{OPERATION}} : 0 \le \lambda \le 1\}. \tag{21}$$

Table S6: Exact V2 group weights. Each row applies to every work-center level and every preset.

Job level j	Instances/group	Operations/group	V_{INSTANCE}	$V_{\text{OPERATION}}$
10	5	250	1/36	1/162
20	5	500	1/36	1/81
50	5	1250	1/36	5/162
100	5	2500	1/36	5/81

$V_{\text{OPERATION}}$ is operation-count exposure. Because every job has five operations, it is algebraically identical to job-count weighting on this corpus. It is not workload, machine load, processing-time exposure, computational difficulty, or industrial importance. No additional endpoint or unrestricted 36-group simplex is admitted. The reference role of w_0 does not weaken A1: the same candidate must satisfy the frozen support rule at both V_{INSTANCE} and $V_{\text{OPERATION}}$.

S1.12 Scientific provenance note

The protocol, design definitions, and reported results in this supplement are fixed scientific records. This presentation introduces no analysis and does not modify them. They remain limited to their declared finite evidence surfaces, matched portfolios, represented uncertainty conditions, and admissible aggregation-design domains; complete end-to-end regeneration of every upstream historical experimental origin is not claimed.

S2. V1 evidence, calibration, and bounded diagnostics

S2.1 Scope and interpretation notice

This supplement section documents the historical/retrospective finite FJSP evidence surface. V1 is retained as a bounded record of what was represented, how the admissible aggregation designs were defined, which deterministic recommendations were obtained, how empirical-resampling variability was calibrated, and why the resulting claim class is A3. The section is an evidence and interpretation record rather than a new analysis.

At each of the three authorized design vertices, MWKR is the unique deterministic point recommendation. That point result must be read together with the controlling empirical-resampling result: the simultaneous-support set is empty at every vertex, including the reference design. MWKR is consequently a deterministic point recommendation only. The final class is `A3 -- Sampling-inconclusive`, meaning inconclusive under the frozen empirical-resampling support calibration, and A3 controls the admissible language throughout S2. The point result is neither erased nor promoted into a supported recommendation.

The scope exclusions are substantive. S2 does not establish algorithm superiority, algorithm equivalence, certification, deployment validity, or a general recommendation for flexible job shop scheduling. It does not enlarge the represented finite benchmark surface into a superpopulation. It also does not compare V1 numerically with V2. In particular, the V1 critical deviation is a surface-specific calibration quantity and is not a cross-surface performance measure.

S2.2 Finite-evidence-surface and group inventory

The V1 target is the observed finite corpus represented by the Brandimarte, DPdata, and Hurink benchmark-family domains. The authorized subset structure comprises Brandimarte–mk, DPdata–dauzere, and the Hurink edata, rdata, sdata, and vdata subsets. Across these six family–subset strata, the surface contains 293 benchmark instances. Each represented subset occurs under nominal, light, and medium processing-time conditions. Their Cartesian inventory forms 18 family–subset–preset groups and 879 instance–preset cells before replication is considered.

The matched comparison portfolio is fixed at five dispatching rules: ECT, FIFO/ECT, SPT, LPT, and MWKR. The cell-level quantity is portfolio-relative and lower is better. A cell is admissible only when all five rules are present for the same instance, preset, and replication identifier. Its denominator is the best positive objective within that exact five-rule cell. If the denominator is nonpositive, the complete cell is excluded for every rule and the failure is recorded; the locked contract reports zero currently excluded cells. These conditions prevent unequal algorithm coverage or denominator substitution from changing the finite surface silently.

Table S7 gives the finite-surface inventory. A replication contributes information about uncertainty for an existing benchmark cell, but it does not create a new benchmark instance or new group mass.

Table S7: Finite V1 evidence-surface inventory.

Field	Definition or value
Represented families	Brandimarte; DPdata; Hurink
Family–subset domains	Brandimarte–mk; DPdata–dauzere; Hurink–edata, rdata, sdata, vdata
Benchmark instances; instance–preset cells; groups	293; 879; 18
Processing-time conditions	nominal; light; medium
Matched portfolio	ECT; FIFO/ECT; SPT; LPT; MWKR
Cell rule	All five rules must be present in the same instance–preset–replication cell; the denominator is the within-cell best positive objective.
Excluded cells	0
Primary replication basis	Identifiers 0–9 uniformly across all groups
Companion basis	Historical mixed basis; descriptive and nonconfirmatory only
Admissible design domain	$\mathrm{conv}\{V_{\mathrm{MICRO}}, V_{\mathrm{EQUAL\_SUBSET}}, V_{\mathrm{EQUAL\_FAMILY}}\}$ (Supplementary Section S1.11)
Reference design	V_{MICRO} on the primary basis

S2.3 Primary and companion replication bases

The primary confirmatory basis is uniform across the full V1 surface. It uses replication identifiers 0–9 in every one of the 18 family–subset–preset groups and is the sole basis for the class-bearing deterministic and

Table S8: V1 deterministic vertex recommendations and simultaneous-support reconstruction.

Vertex	Point recommendation	Simultaneous-support set	Result implication
V_{MICRO}	{MWKR}	empty	Point recommendation only
$V_{\text{EQUAL\_SUBSET}}$	{MWKR}	empty	Point recommendation only
$V_{\text{EQUAL\_FAMILY}}$	{MWKR}	empty	Point recommendation only

Table S9: V1 simultaneous calibration and precedence trace.

Record	Finding and consequence
Resampling	Paired stratified two-way empirical resampling; 20,000 resamples; seed 20260802; 0.95 decision-calibration level.
Family	20 ordered algorithm pairs at each of three vertices: 60 coordinates.
Calibration	Unstudentized directional max-deviation; critical deviation 0.44677028852393696; valid-replicate rate 1.0.
Precedence	A5 does not trigger; A3 triggers because no candidate is supported at the reference design; A1, A2, and A4 are not reached.
Final class	`A3 -- Sampling-inconclusive`.

Table S10: Bounded V1 diagnostics and their status.

Diagnostic	Interpretation
Recommendation and tie frequencies	MWKR frequency 1.0 and tie frequency 0.0 at each vertex describe resampled point outcomes only; neither establishes simultaneous support.
Alternative calibrations and marginal intervals	Descriptive sensitivity material only; cannot replace the complete ordered-60 rule.
Boundary and C6	The deterministic boundary is outside the admissible hull; an in-domain distance and active challenger are not applicable. C6 is diagnostic, noncausal, and non-class-determining.
Mixed-basis companion	Descriptive and nonconfirmatory; does not alter the primary class.

empirical-resampling records summarized here. The uniform basis is also the basis of the frozen reference design b0_micro_uniform_0_9, which is the V1 alias of V_MICRO.

A historical mixed-basis companion is retained for authority and basis-sensitivity inspection. It does not replace the primary basis and is not class determining. Within that companion, only Hurink|edata|nominal and Hurink|edata|pt_noise_medium include the additional replication identifiers 10–19; the other groups remain on identifiers 0–9. The targeted Hurink extension is explicitly nonconfirmatory. V1 must therefore not be described as uniformly replicated over identifiers 0–19.

The distinction is important for weighting. Extra replication depth may affect the precision with which an already represented benchmark cell is described, but it does not increase the benchmark target mass assigned to the corresponding group. The reference design and the two alternative vertices are defined over benchmark cells and authorized group weights, not over the number of available replications. The companion is consequently reported only as a bounded descriptive record. Its retired historical numerical narrative is not reused in S2, and no companion quantity modifies the primary point recommendations, simultaneous-support sets, or A3 class.

S2.4 Deterministic vertex and boundary record

The admissible aggregation domain is the constrained hull generated by three vertices. V_MICRO assigns equal mass per observed instance–preset cell. V_EQUAL_SUBSET assigns equal mass to each represented family–subset domain and equal mass to its presets. V_EQUAL_FAMILY first equalizes the represented families, then the represented subsets within each family, and then the presets within each subset. These are the complete authorized deterministic design vertices. No unrestricted 18-group simplex is part of V1.

At V_MICRO, the complete five-rule portfolio yields the point recommendation set {MWKR}. The same complete-portfolio point recommendation set is obtained at V_EQUAL_SUBSET and V_EQUAL_FAMILY. These three records establish that the deterministic argmin is unchanged over the vertices of the frozen admissible hull. They do not by themselves state anything about simultaneous resampling-calibrated support.

The deterministic boundary authority reports BOUNDARY_OUTSIDE_B_ADM. No recommendation-changing point is identified within the constrained V1 hull. Because the relevant boundary lies outside that domain, the in-domain boundary distance is null or not defined, and no active admissible challenger exists. The empty active-challenger field is therefore not missing evidence to be imputed; it is the authority-defined consequence of the outside-boundary status.

This geometry finding is bounded twice. First, it applies only to the frozen V1 hull generated by the three named vertices and cannot be extended to an unrestricted aggregation simplex. Second, it is a deterministic point property and cannot substitute for the simultaneous empirical-resampling gate. It neither establishes resampling-calibrated support nor implies universal aggregation stability. Any boundary-linked diagnostic requiring an active in-hull challenger is correspondingly unavailable or not applicable.

S2.5 Empirical-resampling and simultaneous calibration record

The primary procedure is the paired stratified two-way empirical-resampling bootstrap specified by the frozen V1 method contract. The six represented family–subset domains are fixed strata. Within each stratum, instances are sampled with replacement from the observed finite inventory, and ten replication identifiers are sampled with replacement from the uniform primary set 0–9. The same sampled replication sequence is applied jointly to the retained instances, all three presets, and all five algorithms. This pairing preserves the matched-cell comparison and the authorized dependence structure; the design weights remain frozen during resampling.

The calibration uses 20,000 empirical resamples, random seed 20260802, and a 0.95 empirical-resampling calibration level. With five algorithms, the complete directional comparison family contains 20 ordered algorithm pairs. Evaluating those pairs at the three distinct design vertices gives 60 coordinates. For each empirical resample and coordinate, the procedure forms the directional deviation between the locked point margin and its resampling counterpart, takes the maximum over all 60 coordinates, and uses the 0.95 quantile of those maxima. The resulting unstudentized directional critical deviation is 0.44677028852393696. The valid-replicate rate is 1.0.

The common critical deviation belongs to this complete V1 surface and comparison family. It must not be interpreted as an effect size, a penalty, or a quantity that can be compared numerically with the calibration of another surface. Reduced comparison families, coordinate-wise marginal intervals, and component resampling variants are retained only as diagnostics. They cannot replace the prespecified ordered-60 family in the claim-bearing reconstruction.

S2.6 Support reconstruction

Table S8 reconstructs the decision at all three vertices using the complete simultaneous family. The point recommendation set is {MWKR} at each vertex, but the candidate-relevant MWKR-versus-ECT simultaneous resampling-calibrated lower bound is nonpositive at each vertex under the common ordered-60 calibration. As a result, MWKR does not satisfy the requirement of a positive simultaneous resampling-calibrated lower bound against every alternative. The simultaneous-support set is therefore empty at `V_MICRO`, `V_EQUAL_SUBSET`, and `V_EQUAL_FAMILY`.

This reconstruction is the controlling support record. It may not be replaced by marginal intervals, a selected subset of challengers, conditional empirical-resampling recommendation frequencies, point rankings, or a reduced comparison family. Several individual directional comparisons have positive resampling-calibrated lower bounds, and MWKR remains the point winner, but a candidate enters the support set only if the complete simultaneous criterion is satisfied against every alternative. The failed candidate-relevant coordinate is sufficient to keep the set empty. The reference-design conclusion is thus not that another rule is supported; it is that no rule is supported under the locked familywise rule.

S2.7 A3 classification trace

The class rules are evaluated in the frozen precedence order `A5 -> A3 -> A1 -> A2 -> A4`. A5 is the mandatory-evidence failure class. It does not trigger because the deterministic authorities, empirical-resampling configuration, complete simultaneous bounds, validity record, and class artifact are present. The procedure therefore reaches the A3 test. A3 triggers because no candidate is resampling-supported at the reference design `b0_micro_uniform_0_9`. Once A3 triggers, A1, A2, and A4 are not reached. Table S9 records both the calibration fields and this precedence trace.

The semantic content of A3 is deliberately narrow. It does not mean that the five algorithms are equivalent, and it does not disprove MWKR as the deterministic point recommendation. It also does not erase the observed stability of the point argmin over the three vertices. Instead, A3 withholds claim-bearing recommendation language because the prespecified simultaneous support requirement is not met at the reference design. Statements that MWKR is supported, superior, reliable, certified, or generally recommended are therefore outside the V1 claim contract.

The outside-boundary status cannot repair this empirical-resampling conclusion. Deterministic invariance within the frozen hull and simultaneous resampling-calibrated support answer different questions and remain separate gates. The final V1 record is consequently `A3 -- Sampling-inconclusive`, with MWKR retained only as a bounded point recommendation.

S2.8 Bounded diagnostics

The diagnostics in Table S10 are included to make the authority record inspectable without expanding its claims. Conditional empirical-resampling recommendation frequency is descriptive only. The locked empirical-resampling record reports MWKR as the resampled point winner with frequency 1.0 at each vertex and a tie frequency of 0.0, but these quantities do not encode a simultaneous resampling-calibrated lower-bound requirement and therefore do not alter the empty support sets. Similarly, marginal percentile intervals and reduced-family calibrations can describe coordinate behavior or calibration sensitivity, but they cannot replace the complete ordered-60 decision.

Boundary summaries also remain subordinate to the deterministic and simultaneous authorities. The bootstrap boundary-inside frequency is descriptive; it does not create an in-hull point boundary, define a boundary distance, identify an active challenger, or establish support. Because the locked point boundary is outside the admissible hull, the boundary distance and active-challenger fields are reported as `NOT_APPLICABLE`. The corresponding C6 boundary-movement decomposition is likewise not applicable rather than numerically reconstructed.

C6 quantities are diagnostic, noncausal, and non-class-determining. Reference-design group contributions are available as signed descriptive decompositions, but no active in-hull challenger exists to provide a claim-bearing boundary link. The C6 gate therefore fails. The instance-only and replication-only calibration records may be used to describe sensitivity to resampling components, but neither component procedure substitutes for the paired primary bootstrap or changes A3. The historical mixed-basis companion has the same bounded status: available for descriptive authority inspection, nonconfirmatory, and excluded from class assignment.

Unavailable or undefined quantities are stated as `UNAVAILABLE`, `NOT_APPLICABLE`, or the equivalent authority status. S2 does not reconstruct a value when the controlling record does not define one. This rule is especially important for the absent boundary distance, active challenger, and boundary-movement contributions.

S2.9 Scientific provenance and reproducibility qualification

V1 has a historical evidence chronology and a later authority lock. The chronology does not create a quality ranking against another surface; it identifies when the finite evidence was produced and when its admissible interpretation was fixed. Conflicting historical numerical material is superseded and excluded from this supplement. The provenance record supports inspection of the frozen protocol and results, but it does not support a complete end-to-end rerun claim.

S2.10 Source and version note

All numerical fields in S2 are fixed evidence records and are displayed without recomputation. The primary class-bearing basis is uniformly replication identifiers 0–9. The historical mixed-basis companion, including its two Hurink edata groups with additional identifiers 10–19, remains descriptive and nonconfirmatory. The claim lock is A3: MWKR is point-only, and the simultaneous-support set is empty at all three vertices. No numerical cross-surface comparison is made.

S3. V2 source, execution, and conformance provenance

S3.1 Scope

V2 is the independently and prospectively frozen second finite FJSP evidence surface. Its source route, structural design, execution contract, aggregation endpoints, and analysis contract were fixed before outcome-bearing interpretation. "Independently" does not mean statistical independence, external validation, deployment validation, or a hierarchy in which V2 validates V1. The two surfaces remain separate and numerically non-comparable.

S3.2 Source and finite-surface identity

The V2 source is the Behnke–Geiger Work Centers benchmark collection. The represented corpus contains 60 static instances arranged in 12 fixed work-center–job strata: work-center levels 5, 10, and 15 crossed with job levels 10, 20, 50, and 100. Each stratum contains five instances. Crossing these strata with nominal, light, and medium processing-time conditions gives 36 structural-stratum–preset groups. Replication identifiers 0–9 provide repeated evidence; they do not create benchmark target mass.

The fixed five-rule matched portfolio is ECT, FIFO/ECT, SPT, LPT, and MWKR. The V2 aggregation endpoints, reference design, and closed admissible segment are defined exactly in Supplementary Section S1.11. In particular, $w_0 = V_{\text{INSTANCE}}$ was frozen prospectively. This reference role anchors the A3/reference and boundary branches, but does not weaken the A1 requirement that the same candidate be supported at both endpoints.

S3.3 Execution and conformance boundary

The execution record comprises 9,000 matched outcome rows: 60 instances, three processing-time conditions, ten replication identifiers, and five algorithms. Independent conformance checks recorded 9,000 schedule-conformance passes, 9,000 makespan-conformance passes, 300 nominal-degeneracy checks, and zero conformance errors. Expected and observed run identities both equal 9,000; expected and observed matched cells both equal 1,800. These checks establish that the declared finite evidence record conforms to its execution contract. They do not establish solver certification, general FJSP superiority, or field performance.

The nominal condition retains its exact degeneracy rather than introducing synthetic randomness. Within each structural stratum, the resampling contract preserves the joint alignment of retained instances, replication identities, processing-time conditions, and all five portfolio algorithms. The aggregation-design weights remain fixed during empirical resampling.

S3.4 Relation to the V2 claim

V2 uses 20,000 paired stratified two-way empirical resamples with seed 20260802 and the surface-specific 0.95 empirical-resampling decision-calibration level. The complete simultaneous family has 40 ordered coordinates: 20 ordered algorithm pairs at each endpoint. ECT is the point recommendation and the simultaneously supported candidate at both V_{INSTANCE} and $V_{\text{OPERATION}}$. All eight ECT-relevant endpoint lower-bound requirements are strictly positive. The permitted additive/common-calibration segment extension and the deterministic boundary record remain restricted to the declared segment; the boundary status is `BOUNDARY_OUTSIDE_B_ADM`. The class is therefore `A1 -- Supported and aggregation-design-stable` within the stated V2 scope.

S3.5 Reproducibility boundary

The supporting evidence record preserves the frozen V2 protocol, evidence, provenance, and integrity checks. It supports inspection of the stated finite-surface contract and its reported results. It does not redistribute raw third-party benchmark source files or claim complete end-to-end regeneration of every upstream historical experimental origin. Memory telemetry is `UNAVAILABLE`; this means unavailable, not measured zero, and is not a scientific validity gate.

S4. Complete Simultaneous-Coordinate Evidence

S4.1 Scope and no-comparison notice

S4 is the reader-auditable coordinate appendix for the frozen simultaneous-support decisions. Its purpose is to expose the complete ordered families that actually enter the two surface-specific support protocols, rather than a selected set of favorable comparisons. For a five-algorithm portfolio, support of a candidate at a fixed design is a complete-portfolio statement: the candidate must satisfy the frozen one-sided simultaneous lower-bound rule against each of its four challengers. The authority family is nevertheless larger than those four candidate-relevant comparisons because every ordered algorithm pair at every required design is retained in the common simultaneous calibration. Reporting all coordinates therefore allows a reader to reconstruct the source family, identify the candidate-relevant subset without deleting any other coordinate, and verify the support set against the same frozen evidence that controlled classification.

Point ranking and simultaneous support remain different objects. A positive point margin, or even a positive simultaneous lower bound for an individual ordered coordinate, does not by itself place that coordinate's first algorithm in a surface support set. Candidate support requires the complete frozen challenger portfolio at that design. This distinction is especially important for V1, where individual coordinates have positive lower bounds although the candidate support set remains empty at every authorized vertex.

> **NO-COMPARISON NOTICE.** V1 and V2 are separately scoped applications of the same claim-bounded protocol and produce different admissibility statuses. Their coordinate values, critical deviations, margins, lower bounds, declared finite evidence surfaces, and aggregation-design geometries are not on an authorized common numerical interpretation scale. No value in one block is to be subtracted from, divided by, normalized against, ranked against, plotted with, or otherwise numerically interpreted through a value in the other block.

The two numerical blocks are therefore physically separated and carry independent calibration provenance. S4 does not treat the surfaces as repeated measurements of one population and does not create a pooled coordinate table, shared axis, ratio, delta, or common magnitude ranking. It is an evidence-reporting section only. It does not establish solver superiority, certification, deployment validity, a universally preferred FJSP rule, or an inference to an unrepresented manufacturing population. These limits follow the locked claim language and finite-scope controls, including the distinction between portfolio-relative evidence and any broader algorithm claim.

S4.2 V1 surface and calibration contract

The V1 block is the authority-locked historical finite evidence surface on the primary `uniform_validated_0_9` basis. Its matched portfolio contains ECT, FIFO/ECT, SPT, LPT, and MWKR. The frozen aggregation-design family contains three distinct vertices: `V_MICRO`, `V_EQUAL_SUBSET`, and `V_EQUAL_FAMILY`. With five algorithms, each vertex retains all 20 ordered algorithm pairs, yielding the controlling 60-coordinate simultaneous family. The reference design identifier `b0_micro_uniform_0_9` is retained in the coordinate authority and aliases the `V_MICRO` vertex rather than creating a fourth family member.

The primary empirical-resampling record is the paired stratified two-way empirical-resampling bootstrap with 20,000 empirical resamples, seed 20260802, a 0.95 empirical-resampling calibration level, frozen design weights, and the linear empirical quantile rule. The familywise construction is the unstudentized directional max-deviation procedure over the complete ordered-60 family. Its frozen V1 critical deviation is `0.44677028852393696`. MWKR is the point recommendation at all three vertices, but the simultaneous-support set is empty at every vertex. The claim-bearing V1 result is therefore `A3 -- Sampling-inconclusive`, meaning inconclusive under the frozen empirical-resampling support calibration. The point result remains a genuine deterministic result, but it is point-only under the frozen complete-family support rule.

S4.3 Complete V1 ordered-coordinate evidence

Table S11 reproduces every row of the controlling V1 simultaneous-coordinate authority. The table preserves the source basis, source design identifier, vertex alias, distinct-member field, ordered algorithms, point-margin literal, simultaneous-lower-bound literal, row critical deviation, and source `supported` field. The added identifiers `V1-C001`–`V1-C060` are rendering identifiers only. No source coordinate is replaced, aggregated, rounded, filtered, or reordered. Exact source numerical precision is retained in the publication table, so no abbreviated-display reconciliation rule is required.

Constant V1 coordinate metadata.
Basis: `uniform_validated_0_9`; distinct-member flag: `True`; common critical deviation: `0.44677028852393696`.
Source-design mapping: `V_MICRO` ↔ `b0_micro_uniform_0_9`; `V_EQUAL_SUBSET` ↔ `V_EQUAL_SUBSET`; `V_EQUAL_FAMILY` ↔ `V_EQUAL_FAMILY`.

Table S11: Complete V1 ordered-coordinate evidence. Exact numeric literals are reproduced without rounding from the controlling simultaneous-coordinate authority. Constant basis, source-design mapping, distinct-member flag, and common critical deviation are stated immediately above; the V1-Cxxx identifiers are publication rendering identifiers only.

Render ID	Vertex / alias	Alg. A	Alg. B	Point margin	Simultaneous LCB	Support
V1-C001	V_MICRO	ECT	FIFO/ECT	2.170859010833424	1.724088722309487	True
V1-C002	V_MICRO	ECT	SPT	1.7136053131715594	1.2668350246476225	True
V1-C003	V_MICRO	ECT	LPT	2.166988014155115	1.7202177256311781	True
V1-C004	V_MICRO	ECT	MWKR	-0.047756274232707466	-0.4945265627566444	False
V1-C005	V_MICRO	FIFO/ECT	ECT	-2.170859010833424	-2.617629299357361	False
V1-C006	V_MICRO	FIFO/ECT	SPT	-0.45725369766186463	-0.9040239861858016	False
V1-C007	V_MICRO	FIFO/ECT	LPT	-0.0038709966783088134	-0.4506412852022458	False
V1-C008	V_MICRO	FIFO/ECT	MWKR	-2.2186152850661314	-2.6653855735900684	False
V1-C009	V_MICRO	SPT	ECT	-1.7136053131715594	-2.1603756016954963	False
V1-C010	V_MICRO	SPT	FIFO/ECT	0.45725369766186463	0.010483409137927668	True
V1-C011	V_MICRO	SPT	LPT	0.4533827009835558	0.006612412459618855	True
V1-C012	V_MICRO	SPT	MWKR	-1.761361587404267	-2.208131875928204	False
V1-C013	V_MICRO	LPT	ECT	-2.166988014155115	-2.6137583026790523	False
V1-C014	V_MICRO	LPT	FIFO/ECT	0.0038709966783088134	-0.44289929184562815	False
V1-C015	V_MICRO	LPT	SPT	-0.4533827009835558	-0.9001529895074928	False
V1-C016	V_MICRO	LPT	MWKR	-2.2147442883878226	-2.6615145769117596	False
V1-C017	V_MICRO	MWKR	ECT	0.047756274232707466	-0.3990140142912295	False
V1-C018	V_MICRO	MWKR	FIFO/ECT	2.2186152850661314	1.7718449965421943	True
V1-C019	V_MICRO	MWKR	SPT	1.761361587404267	1.3145912988803299	True
V1-C020	V_MICRO	MWKR	LPT	2.2147442883878226	1.7679739998638855	True
V1-C021	V_EQUAL_SUBSET	ECT	FIFO/ECT	2.2319083808063698	1.7851380922824327	True
V1-C022	V_EQUAL_SUBSET	ECT	SPT	1.8206975523173723	1.3739272637934352	True
V1-C023	V_EQUAL_SUBSET	ECT	LPT	2.4791844944459815	2.0324142059220445	True
V1-C024	V_EQUAL_SUBSET	ECT	MWKR	-0.09041045057921074	-0.5371807391031477	False
V1-C025	V_EQUAL_SUBSET	FIFO/ECT	ECT	-2.2319083808063698	-2.678678669330307	False
V1-C026	V_EQUAL_SUBSET	FIFO/ECT	SPT	-0.4112108284889975	-0.8579811170129344	False
V1-C027	V_EQUAL_SUBSET	FIFO/ECT	LPT	0.24727611363961177	-0.1994941748843252	False
V1-C028	V_EQUAL_SUBSET	FIFO/ECT	MWKR	-2.3223188313855805	-2.7690891199095176	False
V1-C029	V_EQUAL_SUBSET	SPT	ECT	-1.8206975523173723	-2.2674678408413094	False
V1-C030	V_EQUAL_SUBSET	SPT	FIFO/ECT	0.4112108284889975	-0.03555946003493948	False
V1-C031	V_EQUAL_SUBSET	SPT	LPT	0.6584869421286093	0.2117166536046723	True
V1-C032	V_EQUAL_SUBSET	SPT	MWKR	-1.911108002896583	-2.35787829142052	False
V1-C033	V_EQUAL_SUBSET	LPT	ECT	-2.4791844944459815	-2.9259547829699186	False
V1-C034	V_EQUAL_SUBSET	LPT	FIFO/ECT	-0.24727611363961177	-0.6940464021635487	False
V1-C035	V_EQUAL_SUBSET	LPT	SPT	-0.6584869421286093	-1.1052572306525463	False
V1-C036	V_EQUAL_SUBSET	LPT	MWKR	-2.5695949450251923	-3.0163652335491293	False
V1-C037	V_EQUAL_SUBSET	MWKR	ECT	0.09041045057921074	-0.35635983794472625	False
V1-C038	V_EQUAL_SUBSET	MWKR	FIFO/ECT	2.3223188313855805	1.8755485428616434	True
V1-C039	V_EQUAL_SUBSET	MWKR	SPT	1.911108002896583	1.464337714372646	True
V1-C040	V_EQUAL_SUBSET	MWKR	LPT	2.5695949450251923	2.122824656501255	True
V1-C041	V_EQUAL_FAMILY	ECT	FIFO/ECT	2.334817556690695	1.888047268166758	True
V1-C042	V_EQUAL_FAMILY	ECT	SPT	1.9913295457992426	1.5445592572753055	True
V1-C043	V_EQUAL_FAMILY	ECT	LPT	2.9477383838427773	2.5009680953188402	True
V1-C044	V_EQUAL_FAMILY	ECT	MWKR	-0.1536306522527186	-0.6004009407766555	False
V1-C045	V_EQUAL_FAMILY	FIFO/ECT	ECT	-2.334817556690695	-2.781587845214632	False
V1-C046	V_EQUAL_FAMILY	FIFO/ECT	SPT	-0.3434880108914524	-0.7902582994153894	False
V1-C047	V_EQUAL_FAMILY	FIFO/ECT	LPT	0.6129208271520823	0.16615053862814533	True
V1-C048	V_EQUAL_FAMILY	FIFO/ECT	MWKR	-2.4884482089434137	-2.9352184974673508	False
V1-C049	V_EQUAL_FAMILY	SPT	ECT	-1.9913295457992426	-2.4380998343231797	False
V1-C050	V_EQUAL_FAMILY	SPT	FIFO/ECT	0.3434880108914524	-0.10328227763248454	False
V1-C051	V_EQUAL_FAMILY	SPT	LPT	0.9564088380435347	0.5096385495195977	True
V1-C052	V_EQUAL_FAMILY	SPT	MWKR	-2.1449601980519613	-2.5917304865758983	False
V1-C053	V_EQUAL_FAMILY	LPT	ECT	-2.9477383838427773	-3.3945086723667144	False
V1-C054	V_EQUAL_FAMILY	LPT	FIFO/ECT	-0.6129208271520823	-1.0596911156760194	False
V1-C055	V_EQUAL_FAMILY	LPT	SPT	-0.9564088380435347	-1.4031791265674718	False
V1-C056	V_EQUAL_FAMILY	LPT	MWKR	-3.101369036095496	-3.548139324619433	False
V1-C057	V_EQUAL_FAMILY	MWKR	ECT	0.1536306522527186	-0.29313963627121836	False
V1-C058	V_EQUAL_FAMILY	MWKR	FIFO/ECT	2.4884482089434137	2.0416779204194766	True
V1-C059	V_EQUAL_FAMILY	MWKR	SPT	2.1449601980519613	1.6981899095280242	True
V1-C060	V_EQUAL_FAMILY	MWKR	LPT	3.101369036095496	2.654598747571559	True

S4.4 V1 support reconstruction and class relevance

Table S12 reconstructs the V1 support decision at each frozen vertex without reducing the coordinate family. The point recommendation is {MWKR} at `V_MICRO`, `V_EQUAL_SUBSET`, and `V_EQUAL_FAMILY`. For any candidate in the five-algorithm portfolio, four challenger bounds are required at a fixed vertex. The controlling class authority nevertheless records no simultaneously supported candidate at any of the three vertices, and the full 20 ordered coordinates per vertex remain part of the 60-coordinate calibration regardless of which four bounds are candidate-relevant.

Table S12: V1 support reconstruction under the complete simultaneous family. All fields from the frozen reconstruction are retained in a portrait layout.

**Vertex**	**Support evidence**	**Conclusion, class relevance, and authority**
`V_MICRO`	Point rec. set: {MWKR}; sim. support set: ∅; required bounds/candidate: 4; complete-family status: 20/20 ordered coordinates at this vertex, 60/60 retained overall.	Support conclusion: no candidate enters the simultaneous-support set under the frozen complete-portfolio rule. Class relevance: MWKR remains point-only; A3 is preserved. Authority: frozen V1 authority record 09 + frozen V1 authority record 15; frozen V1 authority record 02.
`V_EQUAL_SUBSET`	Point rec. set: {MWKR}; sim. support set: ∅; required bounds/candidate: 4; complete-family status: 20/20 ordered coordinates at this vertex, 60/60 retained overall.	Support conclusion: no candidate enters the simultaneous-support set under the frozen complete-portfolio rule. Class relevance: MWKR remains point-only; A3 is preserved. Authority: frozen V1 authority record 09 + frozen V1 authority record 15; frozen V1 authority record 02.
`V_EQUAL_FAMILY`	Point rec. set: {MWKR}; sim. support set: ∅; required bounds/candidate: 4; complete-family status: 20/20 ordered coordinates at this vertex, 60/60 retained overall.	Support conclusion: no candidate enters the simultaneous-support set under the frozen complete-portfolio rule. Class relevance: MWKR remains point-only; A3 is preserved. Authority: frozen V1 authority record 09 + frozen V1 authority record 15; frozen V1 authority record 02.

The reconstruction preserves two facts that must not be conflated. First, MWKR's deterministic position is real: it is the point recommendation at every frozen V1 vertex. Second, that deterministic position does not clear the complete simultaneous-support requirement. Individual positive coordinate lower bounds do not alter the support-set reconstruction because support is a conjunction over all four challengers for one candidate, not a count of positive coordinates. The final V1 claim therefore remains A3. In the locked language, A3 means that the present evidence does not resolve a supported reference-design recommendation under the frozen simultaneous rule; it does not mean that the algorithms are equivalent, that MWKR has been disproved, or that another algorithm is thereby supported.

S4.5 V1 coordinate provenance note

The V1 coordinates reproduce the frozen point margins, common critical deviation, simultaneous lower bounds, and support fields without rounding or selecting a favorable subset. S2 gives the V1 evidence and calibration narrative. S4 introduces no S5 diagnostic or C6 contribution quantity.

The following V2 block is independently calibrated and must not be numerically compared with the preceding V1 block.

S4.6 V2 surface and calibration contract

The V2 block is the independently and prospectively frozen second finite FJSP evidence surface, based on the 60-instance Behnke–Geiger surface defined by the prospectively frozen V2 contract. Its matched five-algorithm portfolio is ECT, FIFO/ECT, SPT, LPT, and MWKR. The frozen aggregation-design set has two endpoints, `V_INSTANCE` and `V_OPERATION`. Each endpoint retains all 20 ordered algorithm pairs, giving the controlling 40-coordinate family.

The V2 empirical-resampling authority fixes a paired stratified two-way empirical-resampling bootstrap with 20,000 empirical resamples, seed 20260802, frozen endpoint weights, 12 structural strata, and the linear empirical quantile rule. The V2 critical-deviation authority reports `0.07219365095526062`. Its endpoint support summary records ECT as the point recommendation and the simultaneously supported candidate at both endpoints. The final classification authority therefore records `A1 -- Supported and aggregation-design-stable` within the frozen V2 scope. The boundary status remains `BOUNDARY_OUTSIDE_B_ADM`; that status is restricted to the prescribed two-endpoint admissible design segment and is not a universal stability claim.

S4.7 Complete V2 ordered-coordinate evidence

Table S13 reproduces all 40 rows from the controlling V2 simultaneous-coordinate authority. Endpoint identity, ordered algorithm identity, point-margin literal, V2 critical-deviation literal, simultaneous-lower-bound literal, and the source positive-lower-bound field are retained exactly. The identifiers `V2-C001`–`V2-C040` are publication rendering identifiers only. Reverse ordered comparisons and non-ECT candidate comparisons remain in the table; no selected-challenger or candidate-only reduction is made.

Constant V2 coordinate metadata. Common V2 critical deviation: `0.07219365095526062`. The endpoint, ordered algorithm identities, point margin, simultaneous lower bound, and positive-lower-bound field remain explicit for every coordinate.

Table S13: Complete V2 ordered-coordinate evidence. Exact numeric literals are reproduced without rounding from the controlling V2 simultaneous-coordinate authority. The common critical deviation is stated immediately above; the V2-Cxxx identifiers are publication rendering identifiers only.

Render ID	Endpoint	Alg. A	Alg. B	Point margin	Simultaneous lower bound	Positive L.B.
V2-C001	V_INSTANCE	ECT	FIFO/ECT	0.1290936872078663	0.056900036252605674	True
V2-C002	V_INSTANCE	ECT	SPT	0.5908635490063987	0.5186698980511381	True
V2-C003	V_INSTANCE	ECT	LPT	2.749846664864786	2.6776530139095254	True
V2-C004	V_INSTANCE	ECT	MWKR	0.35870697516548994	0.28651332421022935	True
V2-C005	V_INSTANCE	FIFO/ECT	ECT	-0.1290936872078663	-0.2012873381631269	False
V2-C006	V_INSTANCE	FIFO/ECT	SPT	0.4617698617985324	0.38957621084327176	True
V2-C007	V_INSTANCE	FIFO/ECT	LPT	2.62075297765692	2.548559326701659	True
V2-C008	V_INSTANCE	FIFO/ECT	MWKR	0.22961328795762362	0.157419637002363	True
V2-C009	V_INSTANCE	SPT	ECT	-0.5908635490063987	-0.6630571999616593	False
V2-C010	V_INSTANCE	SPT	FIFO/ECT	-0.4617698617985324	-0.5339635127537931	False
V2-C011	V_INSTANCE	SPT	LPT	2.1589831158583874	2.0867894649031267	True
V2-C012	V_INSTANCE	SPT	MWKR	-0.23215657384090876	-0.30435022479616936	False
V2-C013	V_INSTANCE	LPT	ECT	-2.749846664864786	-2.822040315820047	False
V2-C014	V_INSTANCE	LPT	FIFO/ECT	-2.62075297765692	-2.6929466286121806	False
V2-C015	V_INSTANCE	LPT	SPT	-2.1589831158583874	-2.231176766813648	False
V2-C016	V_INSTANCE	LPT	MWKR	-2.3911396896992962	-2.463333340654557	False
V2-C017	V_INSTANCE	MWKR	ECT	-0.35870697516548994	-0.43090062612075053	False
V2-C018	V_INSTANCE	MWKR	FIFO/ECT	-0.22961328795762362	-0.30180693891288424	False
V2-C019	V_INSTANCE	MWKR	SPT	0.23215657384090876	0.15996292288564815	True
V2-C020	V_INSTANCE	MWKR	LPT	2.3911396896992962	2.3189460387440355	True
V2-C021	V_OPERATION	ECT	FIFO/ECT	0.17103146156491447	0.09883781060965385	True
V2-C022	V_OPERATION	ECT	SPT	0.6875650525795274	0.6153714016242667	True
V2-C023	V_OPERATION	ECT	LPT	2.9227630738067436	2.850569422851483	True
V2-C024	V_OPERATION	ECT	MWKR	0.5681285902919793	0.4959349393367186	True
V2-C025	V_OPERATION	FIFO/ECT	ECT	-0.17103146156491447	-0.2432251125201751	False
V2-C026	V_OPERATION	FIFO/ECT	SPT	0.5165335910146129	0.4443399400593523	True
V2-C027	V_OPERATION	FIFO/ECT	LPT	2.751731612241829	2.6795379612865684	True
V2-C028	V_OPERATION	FIFO/ECT	MWKR	0.3970971287270648	0.32490347777180417	True
V2-C029	V_OPERATION	SPT	ECT	-0.6875650525795274	-0.759758703534788	False
V2-C030	V_OPERATION	SPT	FIFO/ECT	-0.5165335910146129	-0.5887272419698736	False
V2-C031	V_OPERATION	SPT	LPT	2.235198021227216	2.1630043702719552	True
V2-C032	V_OPERATION	SPT	MWKR	-0.11943646228754812	-0.19163011324280874	False
V2-C033	V_OPERATION	LPT	ECT	-2.9227630738067436	-2.9949567247620044	False
V2-C034	V_OPERATION	LPT	FIFO/ECT	-2.751731612241829	-2.82392526319709	False
V2-C035	V_OPERATION	LPT	SPT	-2.235198021227216	-2.3073916721824768	False

Continued on next page

Table S13 – continued

Render ID	Endpoint	Alg. A	Alg. B	Point margin	Simultaneous lower bound	Positive L.B.
V2-C036	V_OPERATION	LPT	MWKR	-2.354634483514764	-2.426828134470025	False
V2-C037	V_OPERATION	MWKR	ECT	-0.5681285902919793	-0.6403222412472399	False
V2-C038	V_OPERATION	MWKR	FIFO/ECT	-0.3970971287270648	-0.46929077968232547	False
V2-C039	V_OPERATION	MWKR	SPT	0.11943646228754812	0.0472428113322875	True
V2-C040	V_OPERATION	MWKR	LPT	2.354634483514764	2.2824408325595034	True

S4.8 V2 support reconstruction and class relevance

Table S14 reconstructs the two endpoint decisions from the locked support records. At each endpoint the point recommendation set is {ECT}, and the simultaneous-support set is also {ECT}. Because the declared portfolio contains five algorithms, ECT must clear four ordered challenger lower bounds at each endpoint. The locked independent reconstruction records all four required bounds as strictly positive at `V_INSTANCE` and all four as strictly positive at `V_OPERATION`; thus all eight ECT-relevant endpoint support records satisfy the source criterion. That candidate-relevant fact does not narrow the inferential family: all 20 ordered coordinates per endpoint and all 40 coordinates overall remain present.

Table S14: V2 endpoint support reconstruction under the complete simultaneous family. All fields from the frozen reconstruction are retained in a portrait layout.

Endpoint	Support evidence	Class relevance and authority
V_INSTANCE	Point rec. set: {ECT}; sim. support set: {ECT}; required ECT bounds: 4; all four positive: True; complete-family status: 20/20 ordered coordinates at this endpoint, 40/40 retained overall.	Class relevance: ECT satisfies the complete support rule; scope-bounded A1 is preserved. Authority: frozen V2 downstream authority 05/06; frozen V2 downstream authority 01/02.
V_OPERATION	Point rec. set: {ECT}; sim. support set: {ECT}; required ECT bounds: 4; all four positive: True; complete-family status: 20/20 ordered coordinates at this endpoint, 40/40 retained overall.	Class relevance: ECT satisfies the complete support rule; scope-bounded A1 is preserved. Authority: frozen V2 downstream authority 05/06; frozen V2 downstream authority 01/02.

The reverse ECT comparisons and the comparisons whose first algorithm is not ECT are retained because the simultaneous family was frozen as an ordered complete family, not as a post-result ECT-versus-challenger subset. The endpoint support summary and independent reconstruction agree on {ECT} at both endpoints, and the final classification authority records A1 within the frozen finite-surface and design-segment scope. The outside-boundary status is part of that bounded record; it does not license a claim about weights or operating environments outside the admissible V2 segment.

S4.9 V2 coordinate provenance note

The V2 coordinates reproduce the frozen point margins, common critical deviation, simultaneous lower bounds, and support fields without rounding or selecting a favorable subset. S3 gives the V2 provenance narrative. No S5 diagnostic quantity is imported here.

S4.10 Scientific provenance note

All V1 and V2 numerical values displayed in S4 are frozen evidence records; the complete 60- and 40-coordinate families are preserved. The V1 class remains `A3 -- Sampling-inconclusive`, with MWKR point-only and empty simultaneous-support sets at all vertices. The V2 class remains scope-bounded `A1 -- Supported and aggregation-design-stable`, with ECT supported at both endpoints and `BOUNDARY_OUTSIDE_B_ADM`. No numerical cross-surface comparison, C6 diagnostic interpretation, solver-superiority, certification, deployment, field-validation, universal-FJSP, or complete end-to-end rerun claim is made.

S5. Diagnostic and noncausal contribution material

S5.1 Role of C6

C6 is diagnostic, noncausal, and non-class-determining. Its quantities may describe a frozen margin or, where one exists, a declared aggregation-weight movement. They do not establish a mechanism, feature importance, performance driver, simultaneous support, an A1–A5 class, or a replacement decision rule. No group is ranked as most important, best, worst, or explanatory.

S5.2 V1 diagnostics

For V1, conditional empirical-resampling recommendation frequencies, marginal summaries, reduced-family calibration summaries, and signed reference-margin decompositions are descriptive only. The point recommendation MWKR appears with resampled point-winner frequency 1.0 at each vertex, but this does not alter the empty simultaneous-support sets or the class `A3 -- Sampling-inconclusive`. The deterministic V1 boundary is `BOUNDARY_OUTSIDE_B_ADM`; therefore no admissible boundary distance, active challenger, or boundary-movement contribution is defined. A historical mixed-basis sensitivity record remains nonconfirmatory and cannot alter the primary uniform-basis inference.

S5.3 V2 diagnostics

For V2, group-level point and empirical-resampling contribution summaries are descriptive decompositions of the frozen endpoint margins. They do not explain why ECT is supported, identify a causal feature, rank structural groups, or provide a second proof of `A1 -- Supported and aggregation-design-stable`. The V2 boundary result remains `BOUNDARY_OUTSIDE_B_ADM` within the declared segment; it is not a universal stability statement.

S5.4 Interpretation boundary

Diagnostic availability is not claim-bearing evidence. Conversely, a diagnostic field that is unavailable or not applicable is not silently reconstructed. C6 is evaluated separately from A1–A5 and cannot change the frozen precedence `A5` → `A3` → `A1` → `A2` → `A4`. No numerical V1–V2 comparison is authorized.

S6. Claim interpretation, scientific provenance, and reproducibility boundary

S6.1 Claim-to-evidence interpretation

The manuscript's claims are interpreted through the finite evidence surfaces and protocol presented in S1–S5. Section S1 defines the matched portfolio, support rule, class precedence, and exact aggregation-design domains. Section S2 gives the V1 evidence and calibration record; Section S3 gives V2 source, execution, and conformance provenance; Section S4 gives the complete ordered-coordinate evidence; and Section S5 sets the diagnostic boundary.

The V1 statement is limited to the historical finite surface: MWKR is point-only, the required support set is empty, and the class is `A3 -- Sampling-inconclusive`. The V2 statement is limited to the prospectively frozen finite surface: ECT is supported at both required endpoints, all eight ECT-relevant endpoint lower-bound requirements are positive, and the class is `A1 -- Supported and aggregation-design-stable`. The two surfaces are not pooled and their numerical values are not compared.

S6.2 Scientific provenance

The displayed contracts, aggregation-design definitions, results, coordinate values, and classifications are frozen scientific records. This supplement presents them for direct reader interpretation and does not recompute or revise them. Integrity checks can confirm the identity and consistency of retained records, but they do not establish a new empirical result, solver certification, deployment validity, or a generally preferable scheduling rule.

S6.3 Reproducibility boundary and limitations

Supporting evidence records preserve the protocol, V1/V2 evidence, provenance, and integrity checks. Raw third-party benchmark source files are not redistributed. Complete end-to-end regeneration of every upstream historical experimental origin is not claimed. This boundary does not alter the finite-surface interpretation of the results; it limits only broader claims about full upstream regeneration.

The supplement does not claim a universal FJSP conclusion, a causal explanation, a statistical population inference, field validation, or deployment performance. C6 remains diagnostic, noncausal, and non-class-determining. These limitations apply alongside, not instead of, the stated V1 and V2 results.
